# Congenital microtia patients: the genetically engineered exosomes released from porous gelatin methacryloyl hydrogel for downstream small RNA profiling, functional modulation of microtia chondrocytes and tissue-engineered ear cartilage regeneration

**DOI:** 10.1186/s12951-022-01352-6

**Published:** 2022-03-28

**Authors:** Jianguo Chen, Tianyu Huang, Ruiquan Liu, Chenyu Wang, Haiyue Jiang, Hengyun Sun

**Affiliations:** Plastic Surgery Hospital, Chinese Academy of Medical Sciences and Peking Union Medical College, No. 33 Badachu Road, Shijingshan District, Beijing, 100144 People’s Republic of China

**Keywords:** Adipose-derived stem cells, Engineered exosomes, Microtia, Porous gelatin methacryloyl, Tissue-engineered ear cartilage

## Abstract

**Background:**

Mesenchymal stem cells (MSCs) exosomes were previously shown to be effective in articular cartilage repairing. However, whether MSCs exosomes promote mature cartilage formation of microtia chondrocytes and the underlying mechanism of action remains unknown. Additionally, some hurdles, such as the low yield and unsatisfactory therapeutic effects of natural exosomes have emerged when considering the translation of exosomes-therapeutics to clinical practices or industrial production. Herein, we investigated the roles of human adipose-derived stem cells (ADSCs) exosomes in modulating microtia chondrocytes and the underlying mechanism of action. Special attention was also paid to the mass production and functional modification of ADSCs exosomes.

**Results:**

We firstly used porous gelatin methacryloyl (Porous Gelma) hydrogel with pores size of 100 to 200 μm for 3D culture of passage 2, 4 and 6 ADSCs (P2, P4 and P6 ADSCs, respectively), and obtained their corresponding exosomes (Exo 2, Exo 4 and Exo 6, respectively). In vitro results showed Exo 2 outperformed both Exo 4 and Exo 6 in enhancing cell proliferation and attenuating apoptosis. However, both Exo 4 and Exo 6 promoted chondrogenesis more than Exo 2 did. Small RNA sequencing results indicated Exo 4 was similar to Exo 6 in small RNA profiles and consistently upregulated PI3K/AKT/mTOR signaling pathway. Notably, we found hsa-miR-23a-3p was highly expressed in Exo 4 and Exo 6 compared to Exo 2, and they modulated microtia chondrocytes by transferring hsa-miR-23a-3p to suppress PTEN expression, and consequently to activate PI3K/AKT/mTOR signaling pathway. Then, we designed genetically engineered exosomes by directly transfecting agomir-23a-3p into parent P4 ADSCs and isolated hsa-miR-23a-3p-rich exosomes for optimizing favorable effects on cell viability and new cartilage formation. Subsequently, we applied the engineered exosomes to in vitro and in vivo tissue-engineered cartilage culture and consistently found that the engineered exosomes could enhance cell proliferation, attenuate apoptosis and promote cartilage regeneration.

**Conclusions:**

Taken together, the porous Gelma hydrogel could be applied to exosomes mass production, and functional modification could be achieved by selecting P4 ADSCs as parent cells and genetically modifying ADSCs. Our engineered exosomes are a promising candidate for tissue-engineered ear cartilage regeneration.

**Graphical Abstract:**

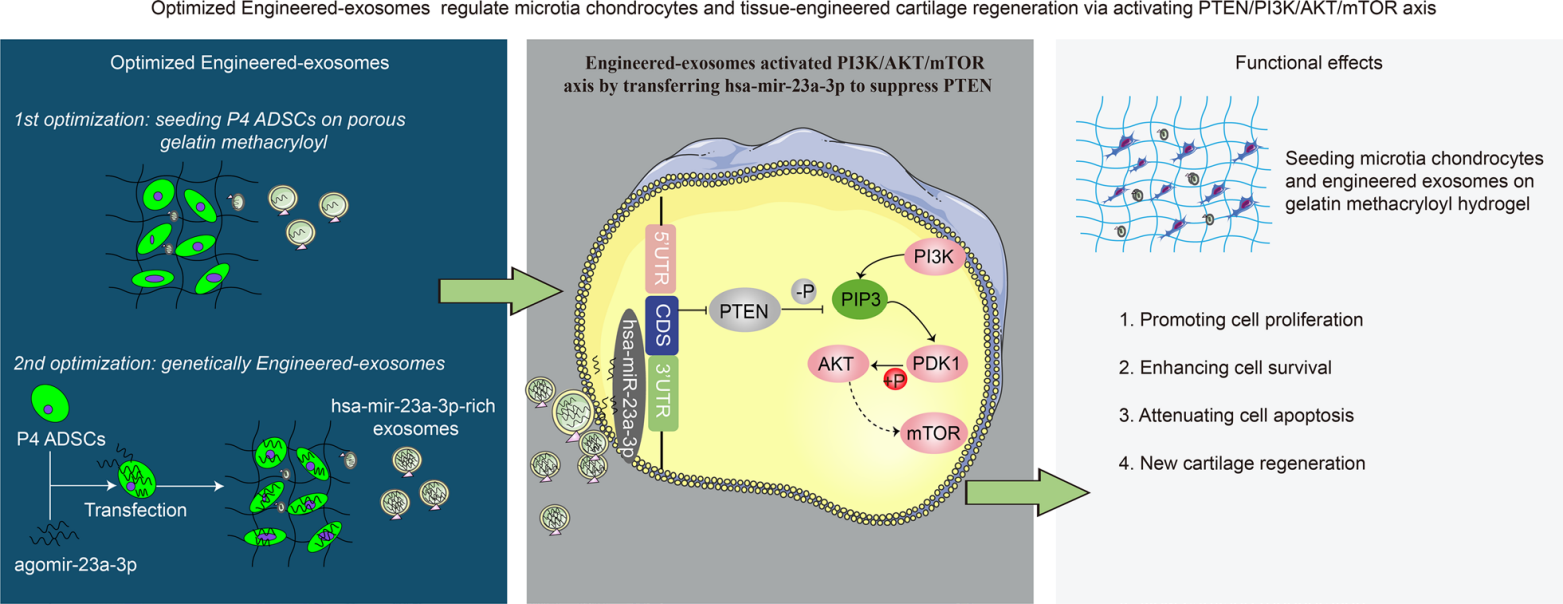

**Supplementary Information:**

The online version contains supplementary material available at 10.1186/s12951-022-01352-6.

## Introduction

With an incidence of 1 to 10 per 10,000 births, microtia is a congenital external ear malformation which will influence the psychological well-being of children [1]. In the past decades, the 7th, 8th and/or 9th autologous rib cartilages have been used widely to reconstruct ears of numerous microtia patients in our ear reconstruction center. Autologous rib cartilage transplantation is the current gold standard treatment for microtia reconstruction, but harvesting rib cartilage inevitably leads to donor site injury and reconstructing the complex ear structure is hard to achieve [2]. The emerging tissue-engineered cartilage provides a novel direction for ear reconstruction [3, 4]. Currently, autologous chondrocytes isolated from microtia cartilage are considered best option as seed cells for constructing tissue-engineered ear cartilage. Our center previously constructed tissue-engineered ear in vitro by seeding microtia chondrocytes onto the 3D-printing biodegradable scaffold [5]. Then the patient-specific ear-shape cartilage frameworks were implanted to reconstruct external ear in five patients. The sufficient cell amount and mature cartilage formation of microtia chondrocytes isolated from a little remnant ear cartilage are the keys to the successful implantation of ear-shape cartilage.

With an average diameter of 100 nm [6], exosomes have been treated either as biomarkers for diagnosis/drug response, or as intercellular communication vehicles to transfer bioactive non-coding RNAs and/or proteins between cells [7–10]. Exosomes released from mesenchymal stem cell (MSCs) were previously shown to be effective in repairing osteochondral/cartilage defects [11–14]. This cell-free therapy could avoid immune rejection and loss of cellular viability that were caused by direct implantation of MSCs for cartilage regeneration [14]. However, whether these exosomes promote mature cartilage formation of microtia chondrocytes and the underlying mechanism of action remains unknown.

Indeed, hurdles have concurrently emerged when considering the translation of exosomes-therapeutics to clinical practices or industrial production [15]. The first one is the low yield, which is mainly attributed to the poor secretion from parent MSCs, the limited culture space and inefficient isolation methods. Some studies have begun to use large cellular platforms to increase yield, such as hollow fiber bioreactors [16, 17], quantum bioreactor culture system [18], spinner flasks bioreactor [19], hyper flasks [20] and microcarrier-based bioreactor culture system [21, 22]. Second, the natural exosomes from parent MSCs often express low-content functional profiles, resulting in unsatisfactory therapeutic outcomes. Some studies have produced engineered exosomes for functional optimization by directly transfecting functional molecules into exosomes [23] or indirectly transfecting functional profiles into parent cells [24, 25]. Lastly, special attention should be paid to the effects of multiple parameters on the exosomal profiles and functions, such as the tissue origin, isolation methods and passage number.

The extracellular vesicles (EVs) derived from ADSCs showed superiority in regenerative potential by promoting the migration, proliferation and chondrogenic/osteogenic differentiation of bone marrow-derived MSCs (BMSCs) as well as in vivo cartilage/bone regeneration, compared with EVs derived from BMSCs or synovium-derived MSCs (SMSCs) [14]. More importantly, in our department of plastic and reconstructive surgery, adipose tissues are more easily obtained from microtia patients undergoing the second stage of ear reconstruction surgery than other tissue origin of MSCs. We hence choose ADSCs as parent cells to produce exosomes, and investigate the effects of ADSCs exosomes on microtia chondrocytes as well as the underlying mechanism of action. With excellent mechanical properties, superior biocompatibility, antibacterial ability and bioactivity, Gelma hydrogel has been widely applied to the field of 3D culture [26] and controlled delivery of bioactive factors [27]. The porous Gelma with reticular pores size of 100 to 200 μm could be favorable to promote ADSCs adhesion/proliferation and nutrients transferring. Indeed, seeding ADSCs on porous Gelma hydrogel helps to lessen culture space and medium volume for producing exosomes. More importantly, it also helps to concentrate exosomes into conditioned medium, consequently increasing the yield of exosomes per volume. Herein we firstly attempt to seed porous Gelma hydrogel (2 mm height; 10 mm diameter) on curing ring for 3D culture of ADSCs, and report the feasibility of releasing exosome in a sustained manner.

Additionally, the International Society for Extracellular Vesicles (ISEV) proposed Minimal Information for Studies of Extracellular Vesicles (MISEV) guidelines in 2014, updated in 2018 [28], for assuring and improving EV research quality. Passage number of parent cells is one of the minimal information for culture condition as the physiological state of parent cells affects the production and cargo composition of exosomes in a passage number-dependent manner. Thus far, to our knowledge, there are no evidence-based studies reporting the effects of passage number of parent cells on the yield, therapeutic small RNA profiles and functional modulation of their corresponding exosomes. To answer these problems, we therefore investigate exosomes derived from P2, P4 and P6 ADSCs as a representative, and aim to draw a conclusion that which passage number would be excellent for cartilage regeneration.

In our study, instead of using large cellular platforms to increase yield, we firstly choose to optimize passage number of parent cells and use porous Gelma hydrogel for 3D culture. It may be useful for increasing yield of exosomes produced per each single cell, reducing culture space and culture medium volume, which subsequently will increase concentration of exosomes per unit volume with minimal costs. We will investigate the feasibility of developing engineered exosomes with high-content specific small RNA by transfecting specific small RNA into parent ADSCs. We mainly evaluate the effects of engineered exosomes on the proliferation, apoptosis and chondrogenesis potential of microtia chondrocytes using tissue-engineered technologies.

## Materials and methods

### Cell isolation and culture

The study was performed strictly in accordance with the guidelines and ethical principles approved by the Ethics Committee of Plastic Surgery Hospital. Written informed consent forms were signed from all individuals. Primary microtia chondrocytes were isolated from microtia patients undergoing the second stage ear reconstructive surgery. According to a previous description [29], ADSCs were isolated from the resected adipose tissues of microtia patients undergoing the second stage ear reconstructive surgery. The details for isolating primary microtia chondrocytes and ADSCs were described at Additional file 1: 1.1. We used human MSC analysis kit (BD Biosciences, USA) to identify the phenotype of cultured ADSCs at passage 2, 4, 6 by flow cytometry analysis.

### Production, isolation and purification of exosomes

Additional file 2 listed the details of ADSCs-cultured parameters for producing exosomes. For 2D-Exo (two dimensional exosomes) production, ADSCs of passage 2 (P2), passage 4 (P4) and passage 6 (P6) were grown in 10-cm dish for 48 h, and then the conditioned medium was collected for 2D-Exo isolation. For 3D-Exo (three dimensional exosomes), P2, P4 and P6 ADSCs were seeded into the porous Gelma hydrogel (EFL-GM-PR-002, Engineering for Life of Yongqinquan, Suzhou, China) and the mixture was loaded into the curing ring (Engineering for Life of Yongqinquan, Suzhou, China). The synthesis of porous Gelma hydrogel composites was described at Additional file 1: 1.2. Then the curing ring together with hydrogel was immersed into 5 ml MSCM for 48 h, and conditioned medium was collected for 3D-Exo isolation. The exosomes were purified from collected conditioned media as previously described [30]. Additional file 3 showed the details of differential ultracentrifuge for exosomes isolation. The concentration/yield were measured using BCA protein assay kit (Beyotime, China, P0010). Freshly purified exosomes were used in our study.

### Exosomes release kinetics and swelling/degradation ratio of porous Gelma hydrogel

Briefly, the porous Gelma hydrogel on the curing ring with 100 µg exosomes were immersed in PBS in 24-well plate. At 1/2/4/8/24/48 h, the surface supernatant was collected and fresh PBS was added. The exosomes released profiles from porous Gelma hydrogel were quantified and expressed as the release percentage as previously described [31]. The 200 µl photo-crosslinked porous Gelma hydrogel dropped on curing ring were soaked in PBS and incubated for 24 h. After removing the surface water, the swelling samples were weighted at 0.5/1/2/4/6/8/24 h. The formula below was used to calculate the swelling ratios: swelling ratio = (W_t_ − W_d_) / W_d_ × 100%. W_d_ represented dry weight of the hydrogel, and W_t_ was the swollen weight of the hydrogel [32]. Meanwhile, we evaluated the degradation behaviors of hydrogel at given time points (day 2, 4, 6, 8, 10, 12, 14, 16). After removing the surface supernatant, the weight of hydrogel was measured. The following formula was used to calculate the degradation ratios: degradation ratio = W_1_/W_0_ × 100%. W_0_ represented the initial wet weight of the hydrogel, and W_1_ was the wet weight of the hydrogel at a given time point (day 2, 4, 6, 8, 10, 12, 14, 16).

### Identification of exosomes: particle size/concentration, morphology and protein markers

According to the Minimal information for studies of extracellular vesicles 2018 (MISEV2018) [28], the exosomes particle size was measured by nanoparticle tracking analysis (NTA) with Zetaview PMX 110 (Particle Metrix, Meerbusch, Germany). The morphology was analyzed by a JEM-1400 transmission electron microscope (TEM) (JEOL, Tokyo, Japan). The protein markers of CD63, CD81, TSG101 and HSP70 were evaluated by Western blotting.

### Study design of exosomes treatment

For in vitro 2D culture, a final concentration of 10 ug/ml exosomes were used. Gelma hydrogel (EFL-GM-30, Engineering for Life of Yongqinquan, Suzhou) were used for in vitro and in vivo culture of issue-engineered cartilage. The microtia chondrocytes seeding density in Gelma hydrogel was 1 × 10^7/ml, and the final concentration of exosomes was 0.5 ug/ul. The blank control was given equivalent volume of PBS.

### Cellular uptake of exosomes

PKH-26 kit (Sigma, USA, PKH26GL-1KT) was used to label exosomes according to the manufacturer’s protocol. The microtia chondrocytes were labelled with carboxyfluorescein diacetate succinimidyl ester (CFDA SE) (Beyotime, China, C1031) according to the manufacturer’s protocol. The labelled exosomes were added into the culture dish or mixed together with Gelma hydrogel and microtia chondrocytes for 24 h at the same culture dish or hydrogel. The details of labelling could be seen at Additional file 1: 1.3. We used the confocal microscopy imaging to observe the uptake of exosomes for the 2D or 3D culture.

### Cell apoptosis assay

Microtia chondrocytes (2 × 10^5) were seeded on 6-well plates and incubated at 37 °C overnight. The monolayer cells were treated with different exposure according to study design for 48–72 h and then were harvested and washed with cold PBS. Cells were re-suspended in binding buffer and stained with Annexin V and propidium iodide (PI) according to manufacturer’s protocol (BD Pharmingen™, USA, 556547). Then the control and treated cells were detected and quantified by flow cytometric analysis. Data were presented as mean ± SD of three number of replicates.

### Cell proliferation assay

The proliferation of microtia chondrocytes was measured by CCK-8 (Dojindo, Japan) according to manufacturer’s protocol. Briefly, 3000 cells were incubated in the 96-well dishes. Then, 10 ul CCK8 was added into each well and co-incubated with cells for 2 h. The cell proliferation was evaluated by 450 nm absorbance values at 0, 24, 48, 72 and 96 h using an enzyme-linked immunosorbent assay plate reader.

### Real-time quantitative PCR (qPCR)

Total RNA was isolated from chondrocytes/tissue-engineered cartilage samples using TRIzol™ reagent (Invitrogen, USA). The RNA was reverse transcribed using PrimeScript™ RT reagent Kit (Takala, RR037A). qPCR was performed in 20-ul reaction volumes using SYBR Green I Master (Roche) according to the manufacture’s protocol. Glyceraldehyde 3-phosphate dehydrogenase (GAPDH) and 5 S rRNA were the internal reference for mRNA and miRNA respectively. The primers were shown in Additional file 4. The related gene expression was calculated using the 2^−ΔΔCT^ method.

### Western blot analysis

Western blotting was performed using standard protocols. Briefly, proteins were denatured, separated on 4–12% polyacrylamide gels, electroblotted, and probed with primary antibody followed by incubation with horseradish peroxidase (HRP)-coupled secondary antibody against the primary antibody. After incubating with the appropriate HRP-coupled secondary antibodies, visualization of the protein bands was performed by incubating with SuperSignal West Pico Chemiluminescent Substrate (Thermo Fisher Scientific). The primary antibodies and secondary antibody used in this study were listed in Additional file 5.

### Small RNA sequencing and analysis

ExoRNeasy Maxi Kit (Qiagen) was used to purify total exosome-derived RNA. After finishing RNA quantification and qualification, a total amount of 3 µg total RNA per sample was used as input material for the small RNA library. The detail of method was described at Additional file 1: 1.4. The significantly differential genes were subjected to Gene Ontology (GO) and Kyoto Encyclopedia of Genes and Genomes (KEGG) analyses [33].

### Luciferase reporter assay and transfection

Transfection was carried out by lipofectamine 3000 reagent (Invitrogen, USA, L3000150) according to the manufacturer’s instruction. The luciferase report vectors were purchased from SyngenTech (Beijing, China). Briefly, luciferase report vectors including *pmir-report-pten wt* and *pmir-report-pten mut* were transfected into HEK-293 t cells with miR-23a-3p mimics or negative control (nc). After 24 h transfection, the cells were cleaved by passive lysis buffer. Firefly and renilla luciferase activity were measured by Dual-Luciferase Reporter Assay System (Promega, USA).

### Treatment of hsa-miR-23a-3p mimics or inhibitor

The hsa-miR-23a-3p mimics/inhibitor and their corresponding nc were purchased from SyngenTech (Beijing, China). Microtia chondrocytes cultured in serum-free medium were treated with 50 nM hsa-miR-23a-3p mimics or its nc (mimics-nc). As a rescue, microtia chondrocytes cultured in serum-free medium were treated with 100 nM hsa-miR-23a-3p inhibitor or its nc (inhibitor-nc). Then, the cells were further cultured in the complete medium.

### Design and assessment of Engineered-Exo

ADSCs cultured in serum-free medium were treated with 50nM agomir-23a-3p or its negative control (agomir-nc). Then, the supernatant was removed and further cultured in the complete medium for 48 h. The transfected P4 ADSCs were seeded on porous Gelma hydrogel for producing Engineered-Exo. The agomir-23a-3p and agomir-nc were purchased from SyngenTech (Beijing, China). Subsequently, quantitative RT-PCR was used to prove the transfection of agomir-23a-3p into ADSCs, and to evaluate the level of hsa-mir-23a-3p within microtia chondrocytes. The protein markers of Engineered-exo and its chondrogenesis potential were also investigated.

### In vitro culture of tissue-engineered cartilage using Engineered-Exo, microtia chondrocytes and Gelma hydrogel

Gelma hydrogel was synthesized as follows: Gelma was first dissolved in 0.1 g/ml PBS (10%) at 50 °C and then was filtered using 0.22 μm filter. The composited hydrogel was obtained after photo crosslinking by 405 nm blue laser radiation for 15 s. Then the hydrogel was immersed in DMEM for in vitro 3D culture.

### In vivo culture of tissue-engineered cartilage and histological evaluation

Animal experiments were approved by Plastic Surgery Hospital Biomedical Ethics Committee. All surgical procedures were performed in accordance with the Guidelines for the Care and Use of Laboratory Animals. A total of 12 male immunodeficient BALB/c nude mice aged 6–8 weeks old were used for the implantation of tissue-engineered cartilage. The nude mice were randomly divided into two groups including: (1) Engineered-Exo; (2) PBS blank control. Briefly, 1 × 10^6 microtia chondrocytes at passage two were harvested and resuspended in 100 µl of 10% Gelma hydrogel (EFL-GM-30, Engineering for Life of Yongqinquan, Suzhou). Next, 100 µg of the freshly purified Engineered-Exo or the equivalent volume of PBS was mixed with the hydrogel. The mixture was dropped into the designed cylindrical mold, and the photocured composited hydrogel was obtained after photo crosslinking by 405 nm blue laser radiation for 15 s. The immunodeficient nude mice were anaesthetized and disinfected. A 0.8-cm median longitudinal incision was made and subcutaneous pockets (1 × 1.5 cm) were designed at the back. The cell-laden Gelma hydrogel was then subcutaneously implanted into the subcutaneous pockets of randomly grouped immunodeficient nude mice. The incision was then closed with suture. Each mouse received one graft. Animals were housed individually and allowed to move without restriction. After 6 weeks, animals were euthanized by CO_2_ inhalation and the samples were harvested for analysis. The tissue-engineered cartilage samples were fixed in 4% paraformaldehyde for 24 h and then the specimens were embedded in paraffin after dehydration in a gradient alcohol. Serial sections were cut at 5-µm and were stained with haematoxylin and eosin (HE) to examine the morphology, along with Alcian blue, Safranin O and Toluidine blue staining to examine collagen matrix deposition surrounding microtia chondrocytes.

### Statistical analysis

Quantitative data were reported as mean ± standard deviation (SD) of three number of replicates. Student’s t-test or one-way analysis of variance (one-way ANOVA) followed by Scheffe’s post-hoc test was conducted for normally distributed data. Mann-Whitney test was conducted for non-normal data. Differences were considered statistically significant when P < 0.05.

## Results

### 2D/3D culture and identification of P2, P4 and P6 ADSCs

**Fig. 1:**
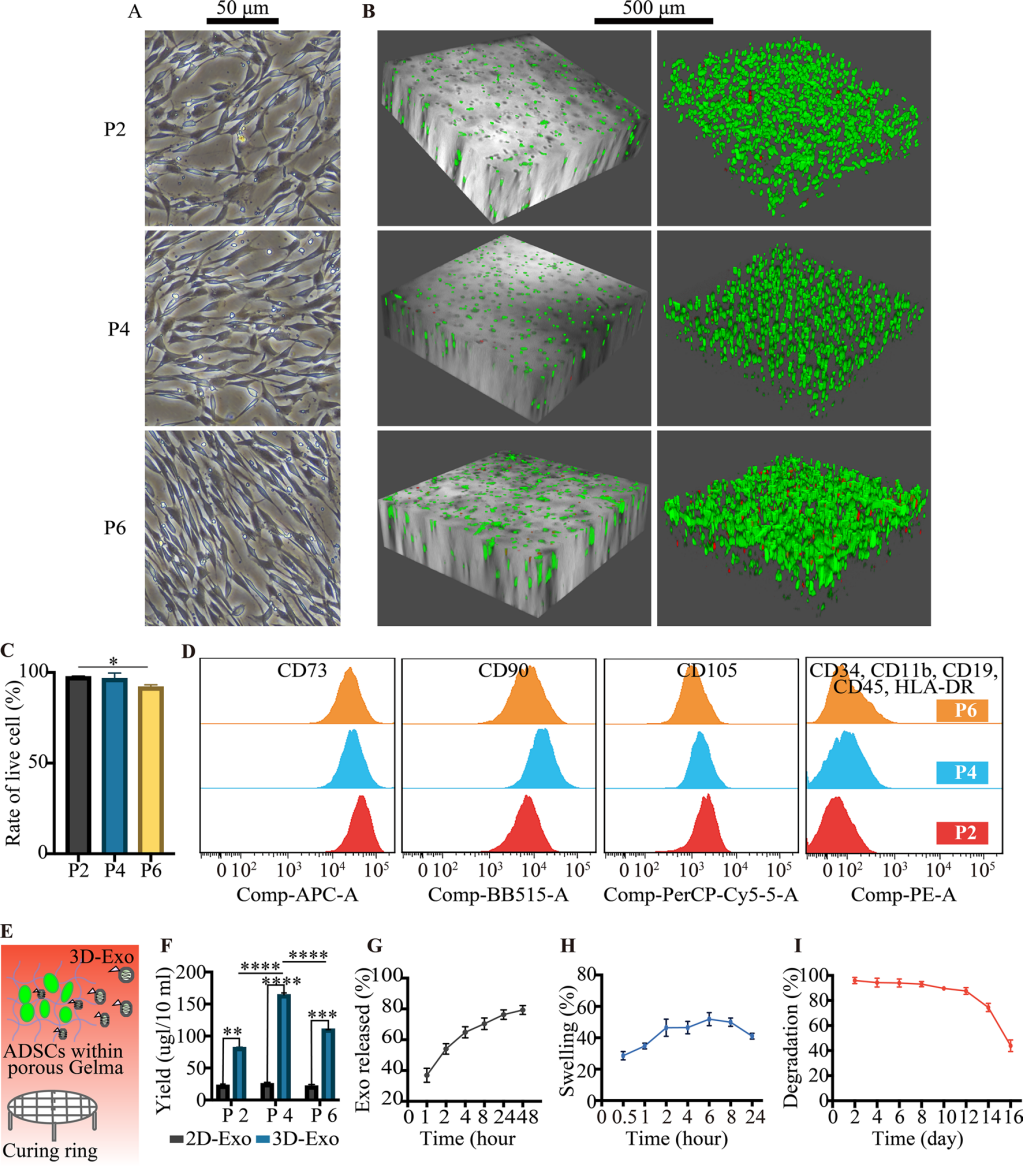
Identification of P2, P4 and P6 human ADSCs and characterization of porous Gelma hydrogel. **A** Representative images showing the spindle-like morphology of ADSCs in 2D culture dish. Scale bar: 50 μm. **B** Representative images showing the live and dead ADSCs within porous Gelma hydrogel, **C** and the rate of live cell. Scale bar: 500 μm. Green: live ADSCs. Red: dead ADSCs. **D** Characteristic surface markers of ADSCs evaluated by flow cytometry analysis. **E** Diagram showing 3D-Exo released from porous Gelma hydrogel. **F** Exosomes yield comparison between 2D- and 3D-cultured conditioned medium from the culture of P2, P4 and P6 ADSCs. **G** Profile of exosomes released from the porous Gelma hydrogel. **H** Swelling ratio of the porous Gelma hydrogel in PBS (pH 7.2) at 37 °C. **I** Degradation ratio of the porous Gelma hydrogel. Data was presented as the mean ± SD of three number of replicates. ∗∗*p* < 0.01, ∗∗∗ *p* < 0.001, ∗∗∗∗*p* < 0.0001

For 2D culture, the P2, P4 and P6 ADSCs morphology observed under optical microscope revealed a spindle-like shape (Fig. 1A). For 3D culture (Fig. 1B and C), live-dead staining assay revealed that P2, P4 and P6 ADSCs remained viable within porous Gelma hydrogel at 48 h, which was especially true for P2 and P4 ADSCs, demonstrating that the hydrogel had good biocompatibility and no cytotoxicity. Flow cytometry analysis revealed P2, P4 and P6 ADSCs were highly positive (> 99%) for MSCs surface markers including CD73, CD90 and CD105 but negative (< 1%) for hematopoietic stem cell (HSC) surface markers CD34 and CD45, which was consistent with the criteria for defining MSCs (Fig. 1D).

### Higher yield of 3D-Exo released from porous Gelma hydrogel

Figure 1E showed the diagram that ADSCs were added into the porous Gelma hydrogel and the hydrogel composites were seeded on curing ring for producing 3D-Exo. The yield of exosomes released from 3D-cultured supernatants was approximately 3.68 to 6.64-fold higher than that from 2D-cultured. 3D-Exo were more concentrated in conditioned medium than 2D-Exo, which could economize culture medium and promote exosomes isolation through labor-intensive ultracentrifugation. For 2D-Exo, disparate passage number of ADSCs showed no significant difference on the yield of exosomes (*P* > 0.05). For 3D-Exo, P4 ADSCs could produce the highest yield of exosomes compared to P2 and P6 ADSCs (*P* < 0.0001) (Fig. 1F). We thus used porous Gelma hydrogel for 3D-Exo mass production in our study.

### Exosomes release kinetics, swelling ratio and degradation ratio of porous Gelma hydrogel

The release kinetics shown a burst release of exosomes from porous Gelma hydrogel within 2 days, corresponding to the time for collecting conditioned medium (Fig. 1G). Due to the porous characteristics, an increase of the swelling ratio during the first 2 h was seen (Fig. 1H). The hydrogel degraded few at the first 12 days and had a noticeable degradation after that. Actually, the degradation ratio could be controlled by changing the crosslink time and precursor solution concentration (Fig. 1I). The results showed porous Gelma hydrogel could be a stable 3D culture material for exosomes mass production.

### Identification of 3D-Exo from disparate passage number of ADSCs

**Fig. 2:**
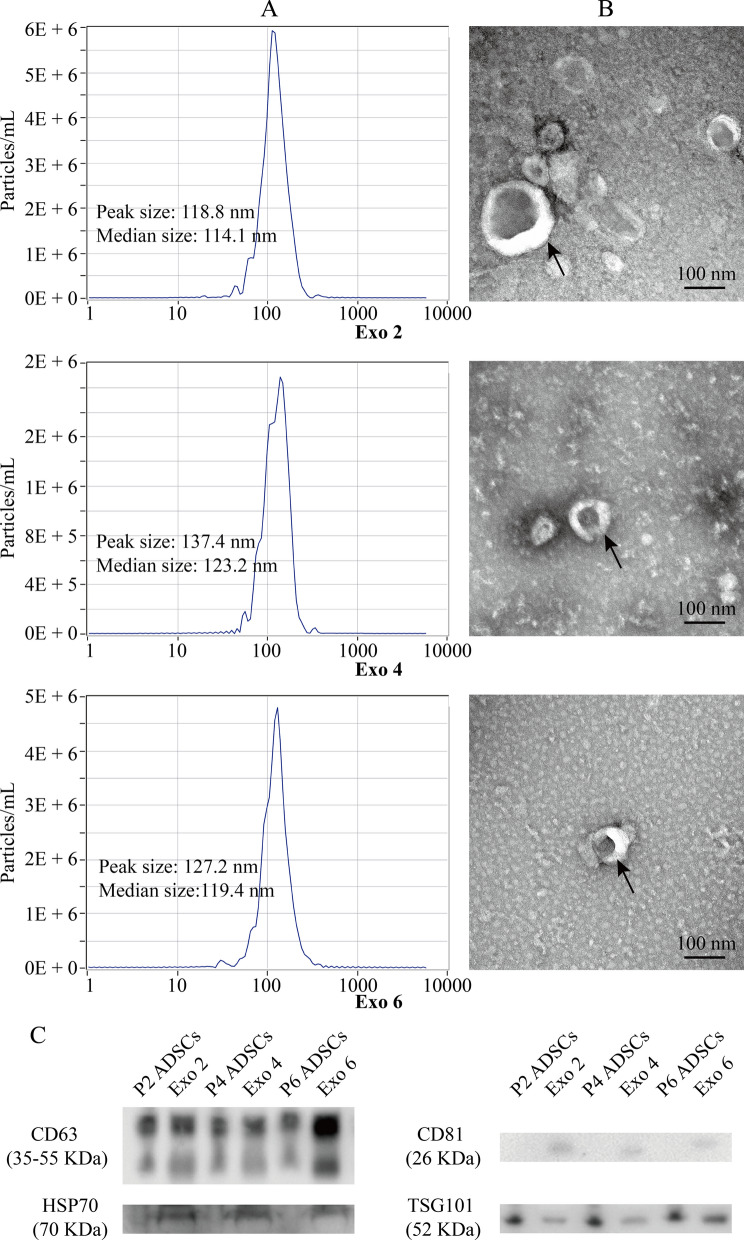
Identification of 3D-Exo released from disparate number of ADSCs (Exo 2, Exo 4 and Exo 6). **A** Particle size distribution measured using nanoparticle tracking analysis. **B** Morphological characterization via transmission electron microscopy. Scale bar: 100 nm. **C** Protein markers of 3D-Exo and their corresponding parental ADSCs extract quantified by western blotting

NTA analysis showed that their median diameters were 114.1 nm, 123.2 nm and 119.4 nm, and their peak diameters were 118.8 nm, 137.4 nm and 127.2 nm, respectively (Fig. 2A). TEM showed that Exo 2, Exo 4 and Exo 6 from 3D culture were bilayer membrane vesicles and their sizes were consistent with NTA analysis (Fig. 2B). Western blotting showed that exosomal markers, including CD63, CD81, HSP70 and TSG101, were expressed in Exo 2, Exo 4 and Exo 6 (Fig. 2C). Additional file 6 shows the original western blotting images. Taken together, 3D-Exo released from porous Gelma hydrogel kept normal morphology without damage.

### The in vitro impact of 3D-Exo on microtia chondrocytes

**Fig. 3:**
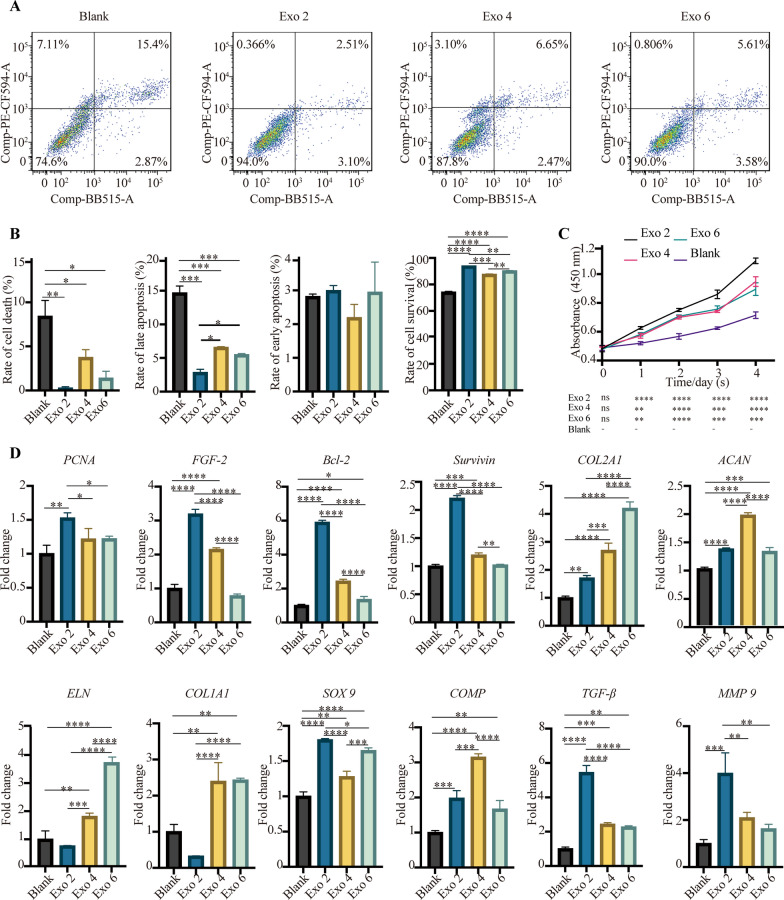
In vitro functional studies showing distinct effects of 3D-Exo on the proliferation, apoptosis and chondrogenesis of microtia chondrocytes. **A** Representative images of cell apoptosis analysis and **B** a quantified comparison of cell early/late apoptosis among groups of PBS control, Exo 2, Exo 4 and Exo 6. **C** Cell proliferation assessed by the Cell Counting Kit-8 at the specific time points after exposure to PBS, Exo 2, Exo 4 and Exo 6. **D** Quantitative RT-PCR analysis showed regulation of genes at 48 h post-treatment associated with anti-apoptosis (*Survivin, Bcl-2*), proliferation (*PCNA, FGF-2*), as well as chondrogenic differentiation and ear cartilage matrix proteins synthesis (TGF-β1, COL2A1, ACAN, ELN, SOX9 and COMP). The genes (COL1A1 and MMP 9) unfavorable for the synthesis of hyaline cartilage and elastic cartilage were also assessed. Data was presented as the mean ± SD of three number of replicates. ∗*p* < 0.05, ∗∗*p* < 0.01, ∗∗∗*p* < 0.001, ∗∗∗∗*p* < 0.0001

We firstly explored the in vitro effects of 3D-Exo on microtia chondrocytes. The experiment design was shown at Additional file 7. We found that 3D-Exo released from disparate passage number of ADSCs exerted distinct effects on microtia chondrocytes. The apoptosis assays showed that 3D-Exo could reduce the programmed cell death, especially reducing the rate of late apoptosis (*P* < 0.001). However, no significant difference was seen in the reduction of early apoptosis (*P* > 0.05). Notably, Exo 2 significantly decreased late apoptosis more than Exo 4 and Exo 6 (*P* < 0.01) (Fig. 3A and B). As shown in Fig. 3C, D-Exo treatment resulted in increased cell proliferation from 24 to 96 h in comparison to blank group. Notably, Exo 2 behaved better than Exo 4 and Exo 6 in enhancing cell proliferation and attenuating apoptosis.

All the above findings were further supported by gene expression analysis (Fig. 3D). Genes associated with cell survival, proliferation and/or apoptosis such as *PCNA, FGF-2, Bcl-2 * and *Survivin* were significantly increased at mRNA levels after exposure to Exo 2 for 48 h compared with PBS group. The mRNA levels of *FGF-2, Bcl-2* and *Survivin* were significantly increased after exposure to Exo 4 compared with PBS group. Nevertheless, no significant difference was seen in the gene expression of *PCNA* between Exo 4 and PBS group. Notably, only the mRNA levels of *Bcl-2* were significantly increased after exposure to Exo 6 compared with PBS group. All in all, we found Exo 2 was more effective to upregulate the expression of the above genes than Exo 4 and Exo 6, corresponding to the results of cell apoptosis and proliferation assays. Nevertheless, compared to Exo 2, Exo 4 and Exo 6 were more effective to upregulate the expression of genes associated with chondrogenic differentiation and cartilage matrix synthesis. Among these genes, we observed significant increase in mRNA levels of chondrogenic factor—TGF-β1 and several ear cartilage matrix proteins-COL2A1, ACAN, ELN, SOX9 and COMP compared to PBS control. The genes such as COL1A1 and MMP 9 are unfavorable for the synthesis of hyaline cartilage and elastic cartilage. We observed that the mRNA level of COL1A1 was significantly increased by the treatment of Exo 4 and Exo 6 but not by Exo 2, compared to PBS control. The mRNA level of MMP 9 was significantly increased by the treatment of Exo 2 but not by Exo 4 and Exo 6, compared to PBS control.

All in all, we observed that Exo 2 was more effective in promoting cell proliferation and reducing late apoptosis in comparison to Exo 4 and Exo 6, but it was less effective than Exo 4 and/or Exo 6 in increasing the mRNA level of cartilage matrix proteins, especially the expression of COL2A1, ACAN, ELN and COMP.

### In vitro 3D-Exo uptake

**Fig. 4:**
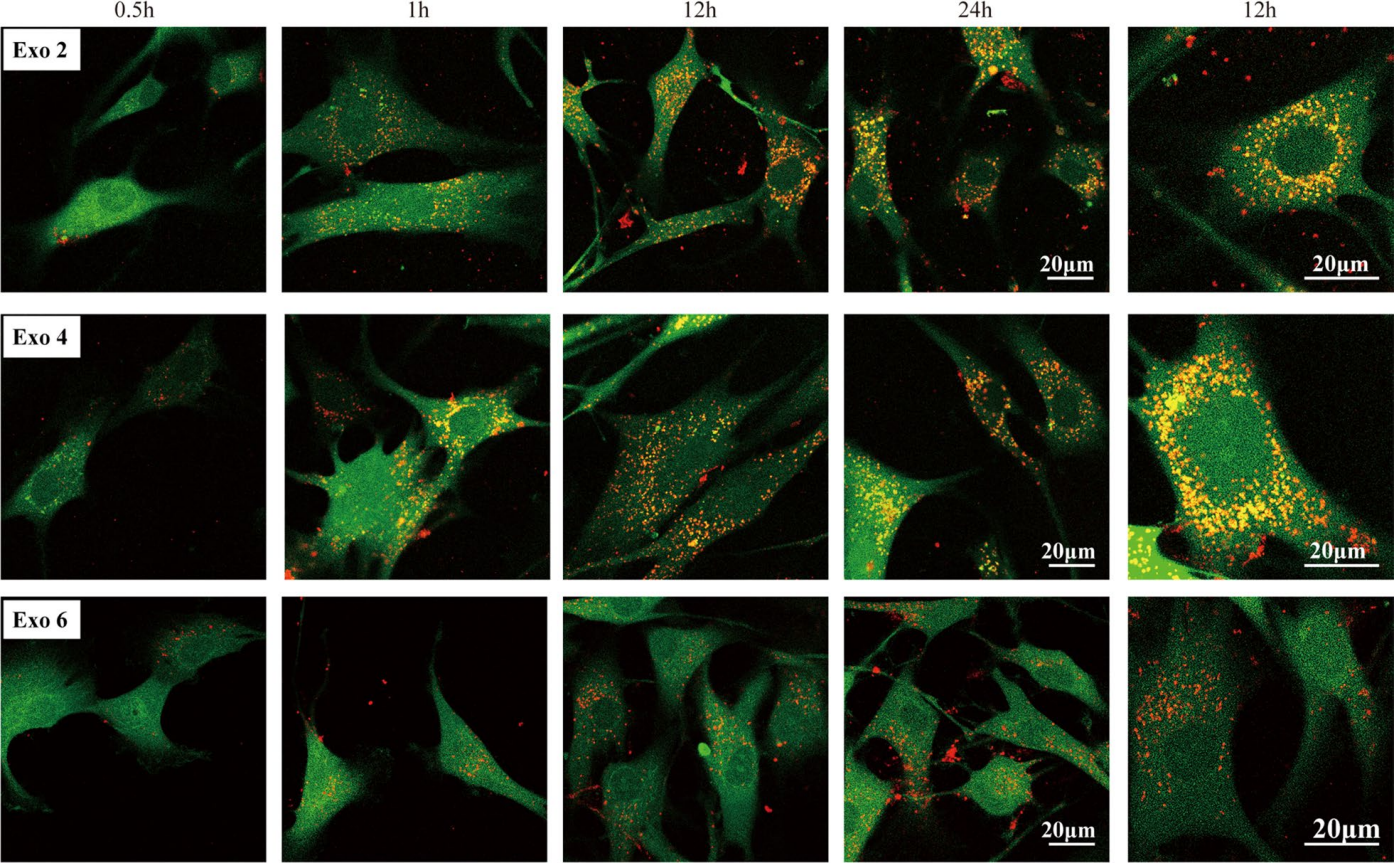
In vitro cellular uptake assay by confocal microscopy imaging demonstrated rapid uptake of 3D-Exo (red). Merged images of CFDA SE-labelled microtia chondrocyte (green) and PKH-26-labelled exosomes (red) at high magnification at 12 h revealed localization of exosomes in the cytoplasm. Scale bar: 20 μm

To assess if 3D-Exo (Exo 2, Exo 4 and Exo 6) interacted directly with microtia chondrocytes, chondrocytes were incubated with labelled 3D-Exo and monitored by confocal microscopy imaging over 24 h. We observed microtia chondrocytes rapidly endocytosed 3D-Exo and became fluorescent within 1 h, and reached a maximum fluorescent intensity from 12 to 24 h. At a higher magnification, the labelled 3D-Exo were found mainly localized in cytoplasm of the cell. These results implied 3D-Exo had the potential to communicate directly and rapidly with microtia chondrocytes (Fig. 4).

### Small RNA sequencing and bioinformatics analysis for Exo2, Exo 4 and Exo 6

**Fig. 5:**
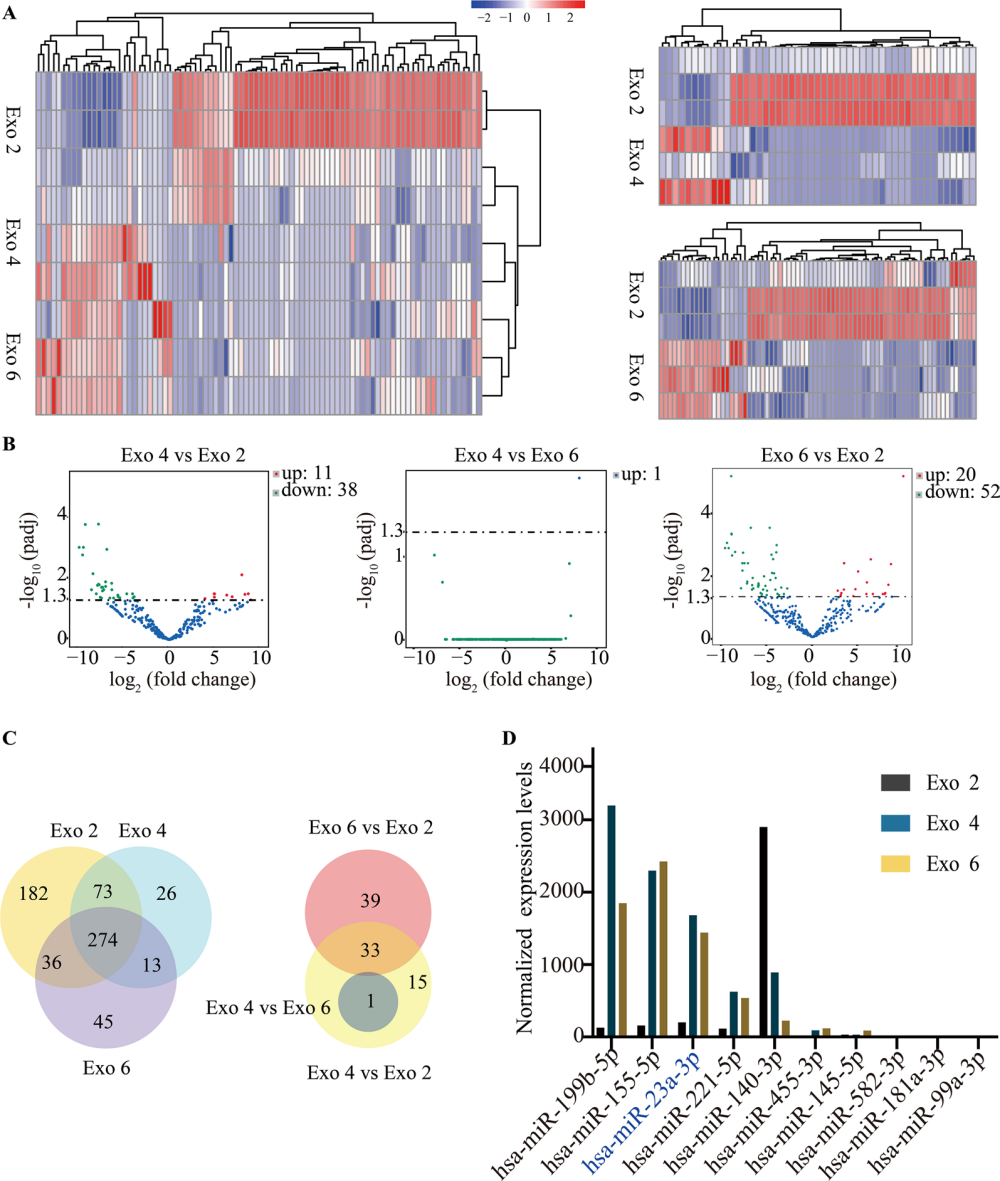
Small RNA sequencing of 3D-Exo. **A** Heat map showing the distinct protein profiles of the Exo 2, Exo 4, and Exo 6. Code from blue (-2 log2 normalized expression) to red (+ 2 log2 normalized expression) indicates RNA expression levels. **B** Volcano plot showing the number of upregulated and downregulated genes in different comparison between two types of 3D-Exo. **C** Venn diagram of unique and shared mRNAs in Exo 2, Exo 4, and Exo 6. **D** Normalized chondrogenesis-related miRNA expression levels

**Fig. 6:**
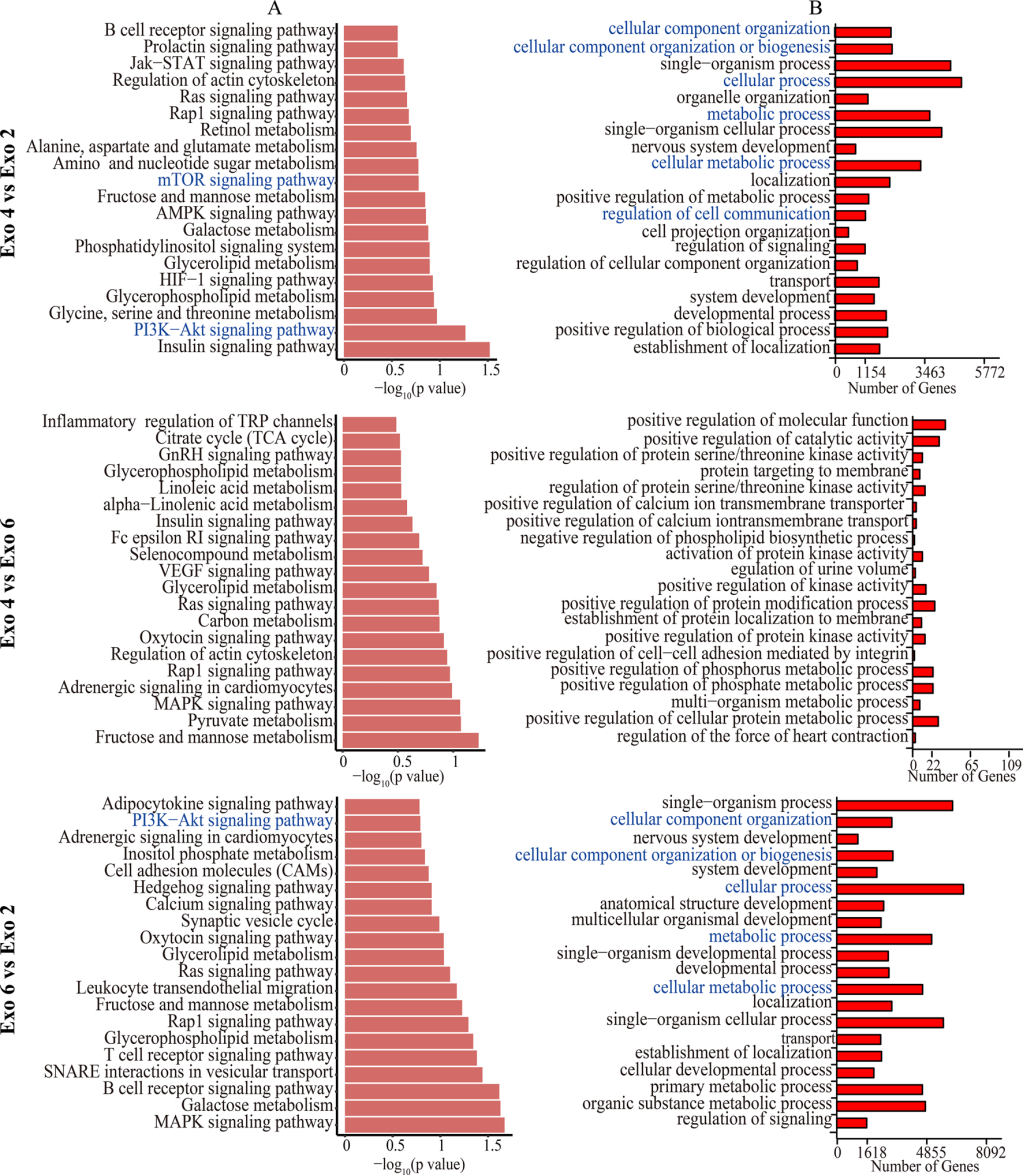
Small RNA sequencing of 3D-Exo revealed the potential mechanism on proliferation, apoptosis and new cartilage regeneration of microtia chondrocytes. **A** Kyoto Encyclopedia of Genes and Genomes enrichment analysis for the upregulated pathways corresponding to the comparisons. **B** Gene ontology analysis for the upregulated biological processes corresponding to the comparisons

Different parameters, such as culture medium, passage number and exosomes isolation methods, would influence the profiles and functions of exosomes. Herein, we firstly detected the impact of passage number on exosomal profiles using small RNA sequencing. The heat map showed biological variations of every type of 3D-Exo in small RNA profiles. As expected in the heat map (Fig. 5A), volcano plot (Fig. 5B) and Venn diagram (Fig. 5C), Exo 4 and Exo 6 were similar in small RNA profiles, but both of them presented distinct profiles compared to Exo 2, indicating that specific passage number affected exosomal cargos. The exosomal cargos related to chondrogenesis were listed in Fig. 5D. Among these various small RNA, hsa-miR-199b-5p, hsa-miR-155-5p and hsa-miR-23a-3p was the first three highly expressed in both Exo 4 and Exo 6 but not in Exo 2, indicating 3D-Exo promoted chondrogenesis probably through delivering hsa-miR-199b-5p, hsa-miR-155-5p and/or hsa-miR-23a-3p. Subsequently, KEGG enrichment pathway analysis (Fig. 6A) revealed that PI3K-AKT and mTOR signaling pathway were consistently upregulated in both Exo 4 and Exo 6 but not in Exo 2. Namely, the small RNA associated with these signaling pathways in Exo 4 and Exo 6 have a higher abundance than those in Exo 2, which is a possible reason for their potential of promoting cell proliferation/growth, reducing cell apoptosis and enhancing cartilage matrix synthesis. Finally, GO analysis (Fig. 6B) revealed that different types of 3D-Exo differentially regulate biological processes, such as cellular component organization or biogenesis, cellular metabolic process, regulation of cell communication and metabolic process.

Subsequently, we investigated which exosomal profile was related to PI3K/AKT/mTOR signaling pathway. PTEN has been known to negatively regulate PI3K/AKT/mTOR signaling pathway by protein dephosphorylation. As for hsa-miR-199b-5p, hsa-miR-155-5p and hsa-miR-23a-3p, the targetscan, Miranda, PicTar, miRmap and PITA databases were used to predict the potential targets. We found PTEN was one of the target genes of hsa-miR-23a-3p (Additional file 8: A). Bioinformatic analysis predicted miR-23a-3p could bind the 3ʹUTR of PTEN (Additional file 8: B).

### 3D-Exo activated the PTEN/PI3K/AKT/mTOR axis of microtia chondrocytes through delivering hsa-miR-23a-3p

Up to now, we have found that Exo 4 and Exo 6 had several advantages when compared to Exo 2: (1) higher yield; (2) better behavior in new cartilage formation; (3) upregulating PI3K/AKT and mTOR signaling pathway. Considering cell senescence and 3D-Exo yield, we thus selected P4 ADSCs for producing 3D-Exo in our study. Subsequently, we investigated whether Exo 4 could activate downstream pathways through delivering miR-23a-3p to suppress PTEN.

**Fig. 7:**
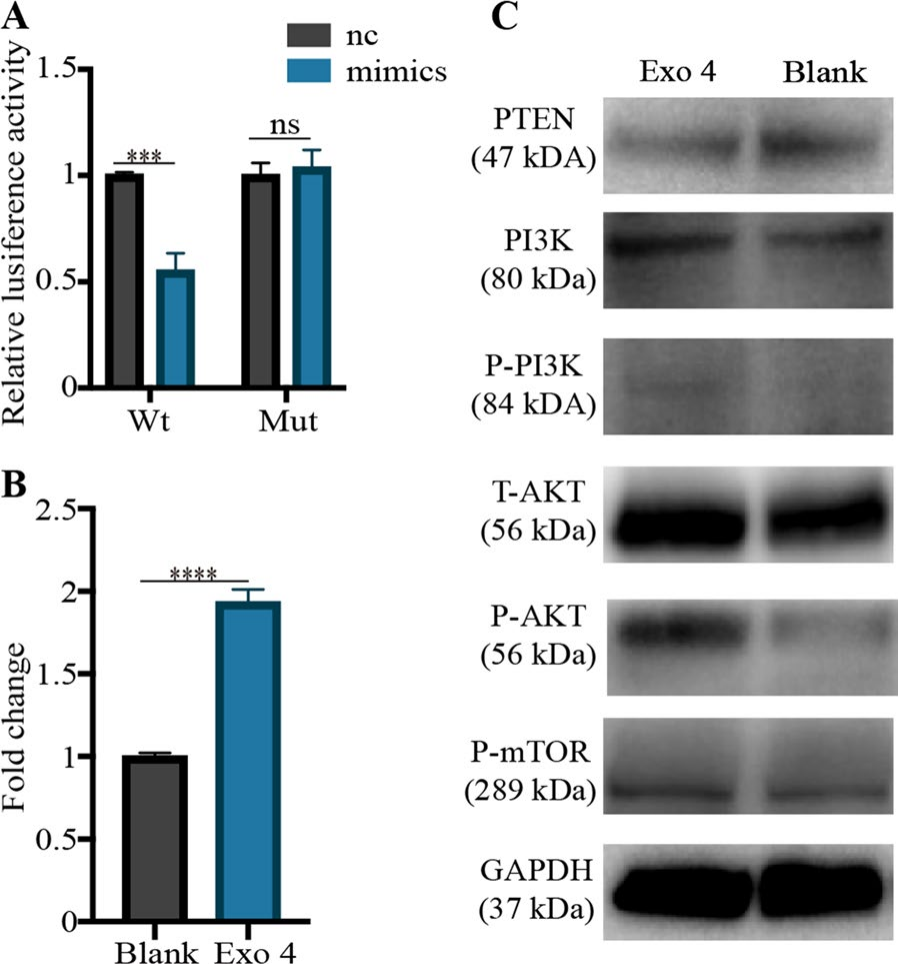
3D-Exo activated the PTEN/P13K/AKT/mTOR signaling pathway by delivering exosomal hsa-miR-23a-3p. **A** The 293 T cells were transfected with luciferase reporter plasmids WT or MUT 3ʹ-UTR of PTEN and hsa-miR-23a-3p mimics/nc, and luciferase activity was detected by Dual-Luciferase Reporter Assay System. **B** Microtia chondrocytes were treated with 3D-Exo for 48 h, then the expression of hsa-miR-23a-3p in microtia chondrocytes was measured by qPCR. **C** Western blotting analysis was used for quantifying the protein levels of PTEN, P-PI3K/PI3K, P-AKT/ AKT, and mTOR in microtia chondrocytes. Data were presented as mean ± SD of three number of replicates. ***P < 0.001, ****P < 0.0001, ns: no significance

Luciferase reporter constructs containing the predicted 3ʹUTR of PTEN and mutated 3ʹUTR of PTEN were cloned and transfected into 293 T cells. When transfected cells were incubated with mimics, the luciferase activity was decreased, and the response was abolished by mutating the target sites in the 3ʹUTR of PTEN. These results confirmed PTEN was the direct target gene of hsa-miR-23a-3p and could be suppressed by hsa-miR-23a-3p (Fig. 7A). After Exo 4 treatment, the level of hsa-miR-23a-3p within microtia chondrocytes was significantly enhanced (*P* < 0.0001) (Fig. 7B). Western blotting revealed that the level of P-PI3K, P-AKT and P-mTOR were significantly increased in Exo 4 group. Additional file 9 shows the original western blotting images. As expected, the level of PTEN was decreased in Exo 4 group (Fig. 7C). Collectively, all results demonstrated that Exo 4 could deliver hsa-miR-23a-3p into microtia chondrocytes. The increased hsa-miR-23a-3p in microtia chondrocytes bound the 3ʹUTR of PTEN and inhibited the expression of PTEN, further negatively regulating the PI3K/AKT/mTOR signaling pathway.

### The essential role of hsa-miR-23a-3p in microtia chondrocytes

**Fig. 8:**
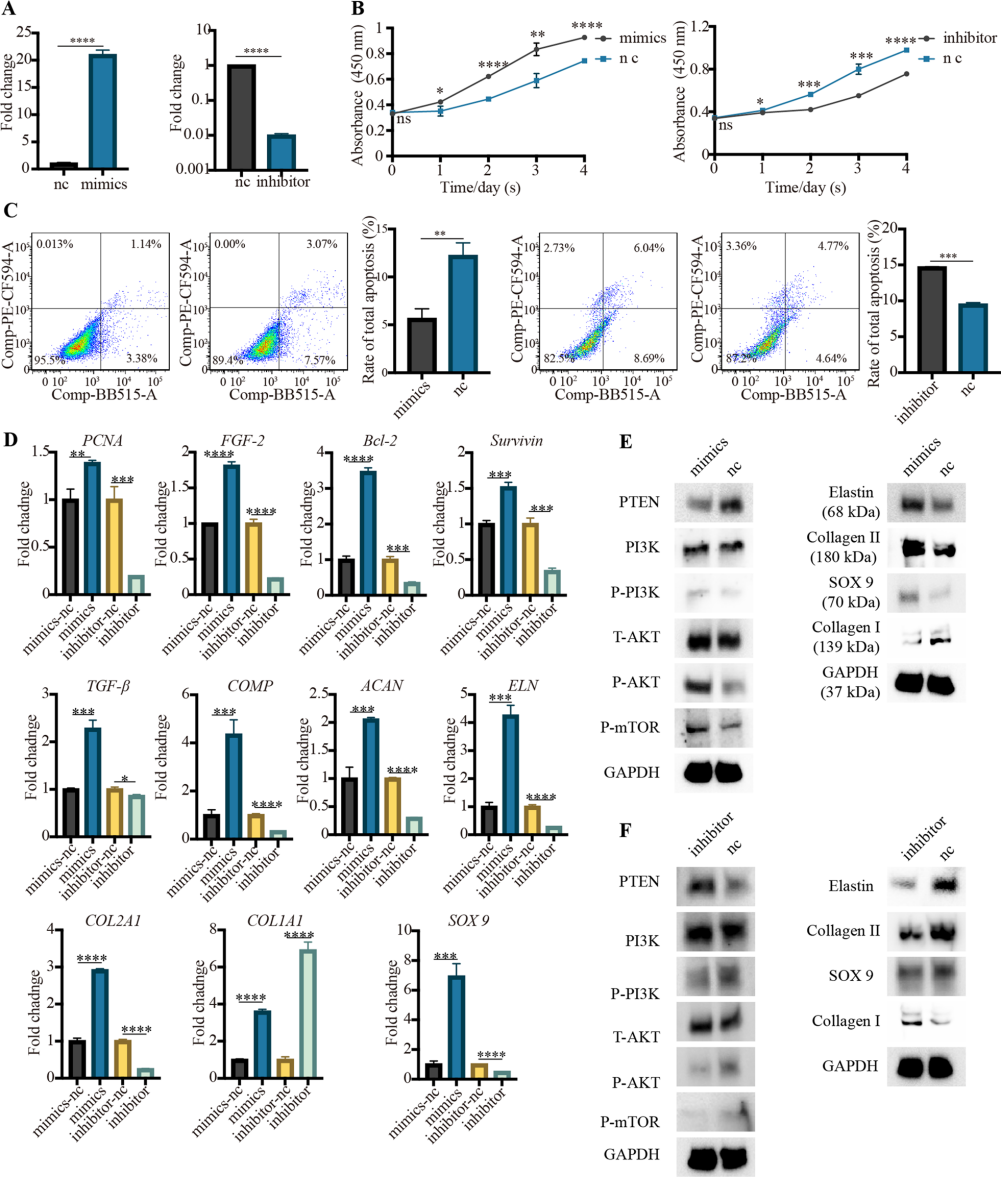
The hsa-miR-23a-3p overexpressing or silencing promoted or attenuated the effect of 3D-Exo on microtia chondrocytes. (**A**) Microtia chondrocytes were treated with hsa-miR-23a-3p mimics/inhibitor or their negative control (nc) for 24 h, and the expression of miR-23a-3p was measured by qPCR. (**B**) Cell proliferation assessed by the Cell Counting Kit-8 after exposure to hsa-miR-23a-3p mimics/inhibitor or their negative control (nc). (**C**) Representative images of cell apoptosis analysis and a quantified comparison of cell early/late apoptosis between the treatment of mimics and nc, or between the treatment of inhibitor and nc. (**D**) Quantitative RT-PCR analysis showed regulation of genes at 48h post-treatment associated with anti-apoptosis (Survivin, Bcl-2), proliferation (PCNA, FGF-2), as well as chondrogenic differentiation and ear cartilage matrix proteins synthesis (TGF-β1, COL2A1, ACAN, ELN, SOX9 and COMP). The genes (COL1A1 and MMP 9) unfavorable for the synthesis of hyaline cartilage and elastic cartilage were also assessed. After exposure to hsa-miR-23a-3p mimics/nc (**E**), or inhibitor/nc (**F**), western blotting analysis was used for quantifying the protein levels of PTEN, P-PI3K/PI3K, P-AKT/ AKT, mTOR and cartilage matrix (i.e. elastin, collagen II, SOX9 and collagen I) in microtia chondrocytes. Data was presented as the mean ±SD of three number of replicates. ∗*p* < 0.05, ∗∗*p* < 0.01, ∗∗∗*p* < 0.001, ∗∗∗∗*p* < 0.0001, ns: no significance

Up to date, the role of hsa-miR-23a-3p in regulating the cellular processes of microtia chondrocytes has not been investigated. As shown in Fig. 8A, the expression of hsa-miR-23a-3p was increased or decreased within microtia chondrocytes when treated with hsa-miR-23a-3p mimics or inhibitor respectively for 48 h. Subsequently, we observed that hsa-miR-23a-3p mimics could promote proliferation (Fig. 8B) and attenuated apoptosis (Fig. 8C) of microtia chondrocytes. Genes associated with chondrocytes survival/proliferation/apoptosis such as *PCNA, FGF-2, Bcl-2* and *Survivin* were significantly increased at mRNA levels within 48 h of exposure to mimics. Besides, hsa-miR-23a-3p mimics robustly enhanced the expression of several genes associated with chondrogenic differentiation and extracellular matrix synthesis. Among these genes, we observed significant increase in mRNA levels of chondrogenic factor—TGF-β1 and several cartilage matrix proteins-COL2A1, ACAN, ELN, SOX9 and COMP compared to the negative control (mimics-nc). However, the mRNA levels of COL1A1 was also increased in hsa-miR-23a-3p mimics group, which was unfavorable for the regeneration of hyaline cartilage and elastic cartilage (Fig. 8D). Western blotting revealed that the level of P-PI3K, P-AKT and P-mTOR in microtia chondrocytes were increased in the presence of mimics, whereas the level of T-AKT and PI3K was not significantly increased. As expected, the level of PTEN was decreased in the presence of mimics (Fig. 8E). Western blotting further proved that hsa-miR-23a-3p mimics could enhance the protein level of elastin, collagen II and SOX9 for cartilage regeneration. Nevertheless, we observed that the protein expression of collagen I was decreased in the presence of mimics, which was inconsistent with the results of qRT-PCR (Fig. 8E). Additional file 10 shows the original western blotting images for the modulation of hsa-miR-23a-3p mimics. The above findings related to the proliferation, apoptosis and chondrogenesis were rescued by hsa-miR-23a-3p inhibitor, which further proved the essential effects of hsa-miR-23a-3p on microtia chondrocytes (Fig. 8B–F). Additional file 11 shows the original western blotting images for the modulation of hsa-miR-23a-3p inhibitor.

### The evaluation of engineered hsa-miR-23a-3p-rich exosomes (Engineered-Exo)

**Fig. 9:**
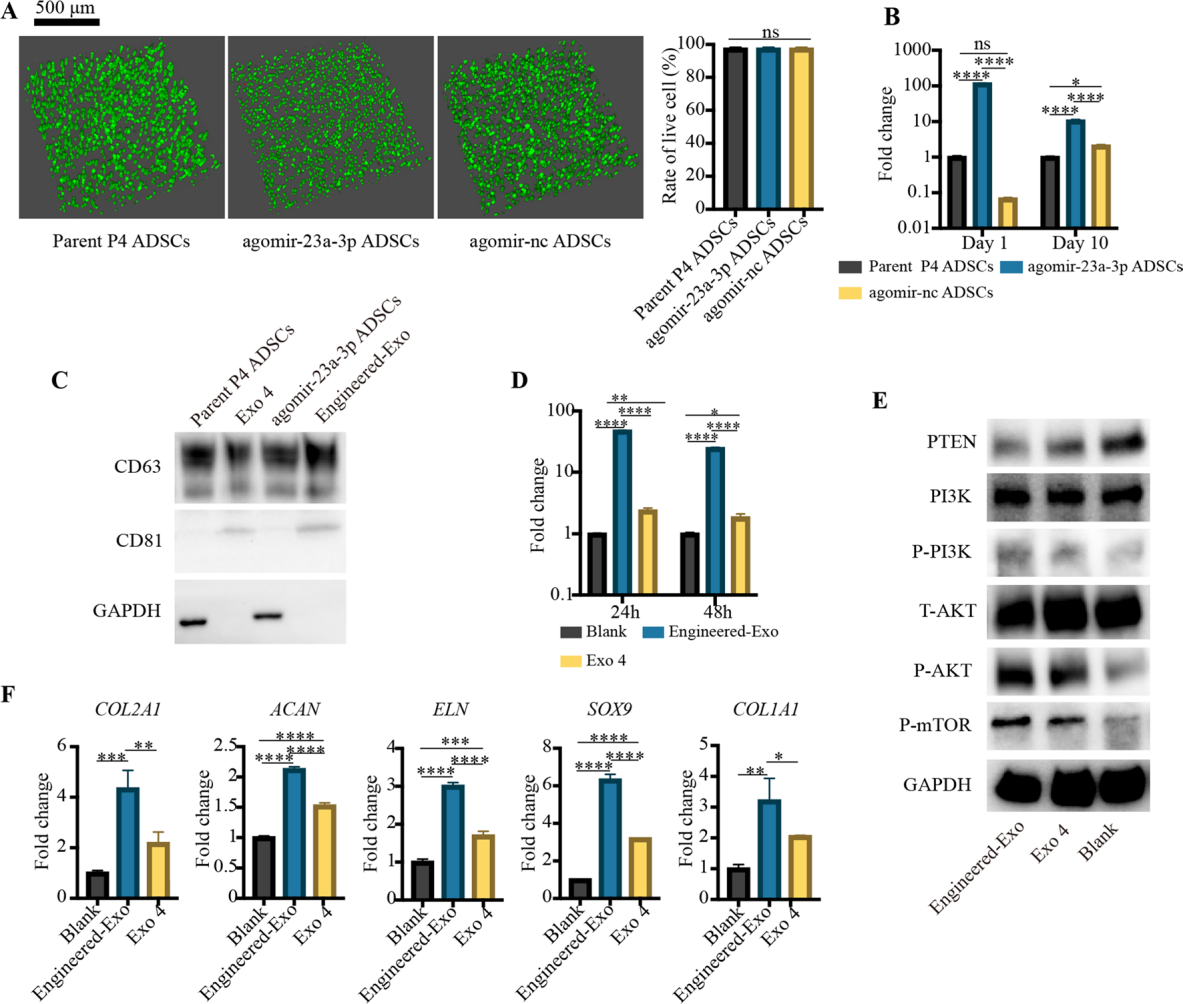
The design of Engineered-Exo. **A** Representative images showing the live and dead ADSCs (Parental ADSCs, agomir-23a-3p ADSCs and agomir-nc ADSCs) within porous Gelma hydrogel, and the rate of live cell. Scale bar: 500 μm. Green: live ADSCs. Red: dead ADSCs. **B** Quantitative RT-PCR analysis showed the level of hsa-mir-23a-3p within three types of ADSCs at 1 day and 10 day after transfection. **C** Protein markers of Engineered-Exo, Exo 4 and their corresponding ADSCs extract quantified by western blotting. **D** Quantitative RT-PCR analysis showed the level of hsa-mir-23a-3p within microtia chondrocytes at 24 and 48 h post-treatment. **E** Western blotting analysis was used for quantifying the protein levels of PTEN, P-PI3K/PI3K, P-AKT/ AKT and mTOR in microtia chondrocytes at 72 h post-treatment. **F** Quantitative RT-PCR analysis showed the mRNA level of COL2A1, ACAN, ELN, SOX9 and COL1A1 within microtia chondrocytes at 48 h post-treatment. Data was presented as the mean ± SD of three number of replicates. ∗*p* < 0.05, ∗∗*p* < 0.01, ∗∗∗*p* < 0.001, ∗∗∗∗*p* < 0.0001, ns: no significance

To load high content of hsa-miR-23a-3p into Exo 4, we decided to design an Engineered-Exo by transfecting agomir-23a-3p into P4 ADSCs (agomir-23a-3p ADSCs). The parent P4 ADSCs (without transfection) and agomir-nc ADSCs were treated as control group. The three types of ADSCs were seeded on porous Gelma hydrogel. Live-dead staining showed that the agomir-23a-3p ADSCs were still remained viable (Fig. 9A), indicating that the transfection of agomir-23a-3p would not affect the survival of 3D-cultured ADSCs. Compared to parent ADSCs, the level of hsa-miR-23a-3p increased 116.67-fold approximately in agomir-23a-3p ADSCs initially, and after 10-day 3D culture, we observed that the content of hsa-miR-23a-3p still had a 10-fold enhancement (Fig. 9B). The purified Engineered-Exo showed higher protein markers of CD63 and CD81 than the natural Exo 4 (Fig. 9C). Additional file 12 shows the original western blotting images***.*** Subsequently, we evaluated the communicating efficiency of Engineered-Exo. Compared to PBS group, both Engineered-Exo and natural Exo 4 from parent ADSCs could enhance the level of hsa-miR-23a-3p of microtia chondrocytes. Compared with Exo 4, Engineered-Exo could increase the levels of hsa-miR-23a-3p of microtia chondrocytes by about 20 times approximately at 24 h, and by about 13 times at 48 h after treatment (Fig. 9D). Compared to Exo 4 and PBS blank control, Engineered-Exo could significantly enhance the level of P-PI3K, P-AKT and P-mTOR of microtia chondrocytes, whereas the level of T-AKT and PI3K was not significantly increased. As expected, the level of PTEN was significantly decreased in the presence of Engineered-Exo (Fig. 9E). Additional file 13 shows the original western blotting images. Additionally, the mRNA level of several ear cartilage matrix proteins, such as *COL2A1*, *ACAN*, *ELN*, *SOX9*, were significantly enhanced in Engineered-Exo and Exo 4 group, but Engineered-Exo did better than Exo 4. However, the mRNA level of *COL1A1* was also increased in both Engineered-Exo and Exo 4 group, which was unfavorable for the regeneration of hyaline cartilage and elastic cartilage (Fig. 9F). All in all, the Engineered-Exo seemed to be more effective to activate the PTEN/PI3K/AKT/mTOR axis and to regenerate cartilage. It would be applied in our center for further studies.

### In vitro tissue-engineered cartilage regeneration: Engineered-Exo, microtia chondrocytes and Gelma bio-ink

**Fig. 10:**
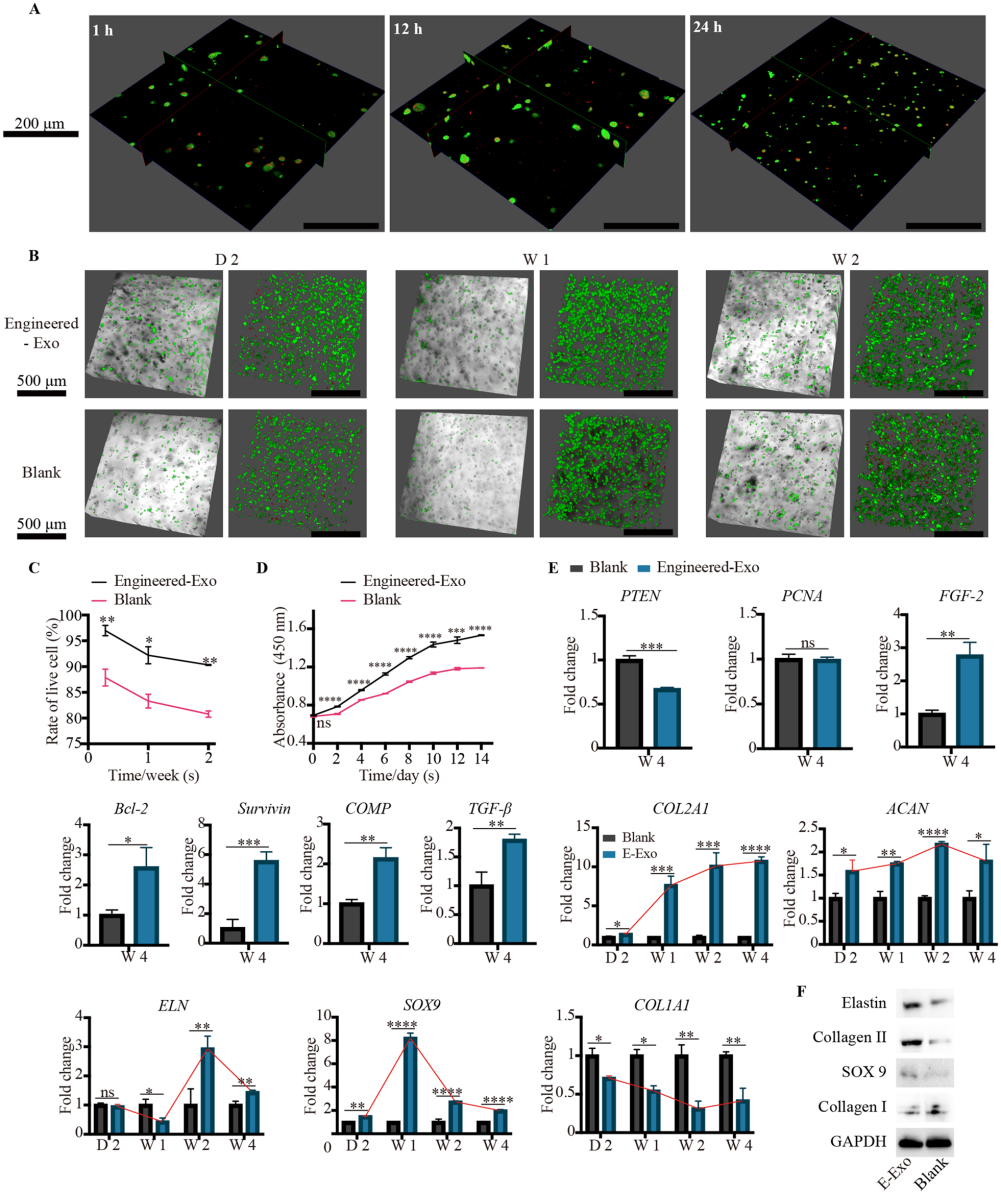
Engineered-Exo promoted the survival, proliferation and new cartilage formation of 3D-cultured tissue-engineered cartilage. (**A**) Cellular uptake assay by confocal microscopy imaging demonstrated rapid uptake of Engineered-Exo (red) at the different time points. (**B**) Representative images showing the live and dead microtia chondrocytes seeded on Gelma hydrogel, and (**C**) the comparison for the rate of live cell between Engineered-Exo and PBS control. Scale bar: 500 μm. Green: live microtia chondrocytes. Red: dead microtia chondrocytes. (**D**) Cell proliferation assessed by the Cell Counting Kit-8 at the specific time points after the exposure to Engineered-Exo or PBS. (**E**) Quantitative RT-PCR analysis showed regulation of genes associated with anti-apoptosis (PTEN, Survivin, Bcl-2), proliferation (PTEN, PCNA, FGF-2), as well as chondrogenic differentiation and ear cartilage matrix proteins synthesis (PTEN, TGF-β1, COL2A1, ACAN, ELN, SOX9 and COMP). The genes (COL1A1 and MMP 9) unfavorable for the synthesis of hyaline cartilage and elastic cartilage were also assessed. In particular, the tendency of expression of several chondrogenesis-related genes (COL2A1, ACAN, ELN, SOX9 and COL1A1) was described. (**F**) The protein levels of elastin, collagen II, and SOX9 in microtia chondrocytes were analyzed by western blotting. Data was presented as the mean ±SD of three number of replicates. ∗*p* < 0.05, ∗∗*p* < 0.01, ∗∗∗*p* < 0.001, ∗∗∗∗*p* < 0.0001

Presently, Gelma hydrogel has been used as 3D printing bio-ink for tissue-engineered cartilage in our center, and herein we would investigate the efficacy of Engineered-Exo for in vitro 3D culture of tissue-engineered cartilage. We used confocal microscopy imaging to assess if Engineered-Exo interact directly with microtia chondrocytes seeded on Gelma hydrogel. As expected, we observed that the cells rapidly endocytosed the Engineered-Exo and became fluorescent within an hour and reached a maximum fluorescent intensity from 12 to 24 h, which was similar to the results of in vitro uptake assays. The labelled exosomes were found mainly localized in cytoplasm of the cell (Fig. 10A).

Live-dead staining revealed that Engineered Exo could significantly enhance the live rate of microtia chondrocytes embedded into Gelma hydrogel in comparison to PBS group after 2 days, 1 week and 2 weeks (Fig. 10B and C). Additionally, Engineered-Exo treatment could significantly increase proliferation of microtia chondrocytes from day 1 to day 14 as evaluated by CCK-8 assays (Fig. 10D). Compared to PBS control, PTEN was significantly decreased at mRNA levels after 4-week exposure to Engineered-Exo, and genes associated with chondrocyte survival/proliferation/apoptosis such as *FGF-2, Bcl-2* and *Survivin* were significantly increased at mRNA levels after 4-week exposure to Engineered-Exo. We also found that Engineered-Exo robustly enhanced the expression of chondrogenic factor—*TGF-β1* and *COMP* after 4-week treatment. In particular, we evaluated the dynamic change of several ear cartilage matrix proteins-*COL2A1, ACAN, ELN, SOX9* and *COL1A1* after 2-day, 1-week, 2-week and 4-week treatment of Engineered-Exo in comparison to the treatment of PBS. As shown, every type of matrix protein shown a disparate tendency of gene expression. *COL2A1* was steadily risen and reached equilibrium stage at week 4. *ACAN* declined after it reached the peak at week 2, while *SOX9* declined after it reached the peak at week 1. The gene expression of *ELN* was not significantly increased at day 2 and even decreased at week 1. Nevertheless, at week 2, the mRNA level was significantly risen and reached the peak. After that, the gene expression began to decline but was still higher than that of PBS control group. As expected, *COL1A1* was steadily declined and reached its bottom at week 3. Though the expression began to incline in week 4, the mRNA level in Engineered-Exo group was still lower than that in blank group (Fig. 10E). As assessed by western blotting, the protein level of elastin, collagen II and SOX9 was significantly increased while collagen I was decreased after 4-week treatment of Engineered-Exo, which was consistent with the results of qPCR (Fig. 10F). Additional file 14 shows the original western blotting images.

### The effects of Engineered-Exo on tissue-engineered cartilage regeneration

**Fig. 11:**
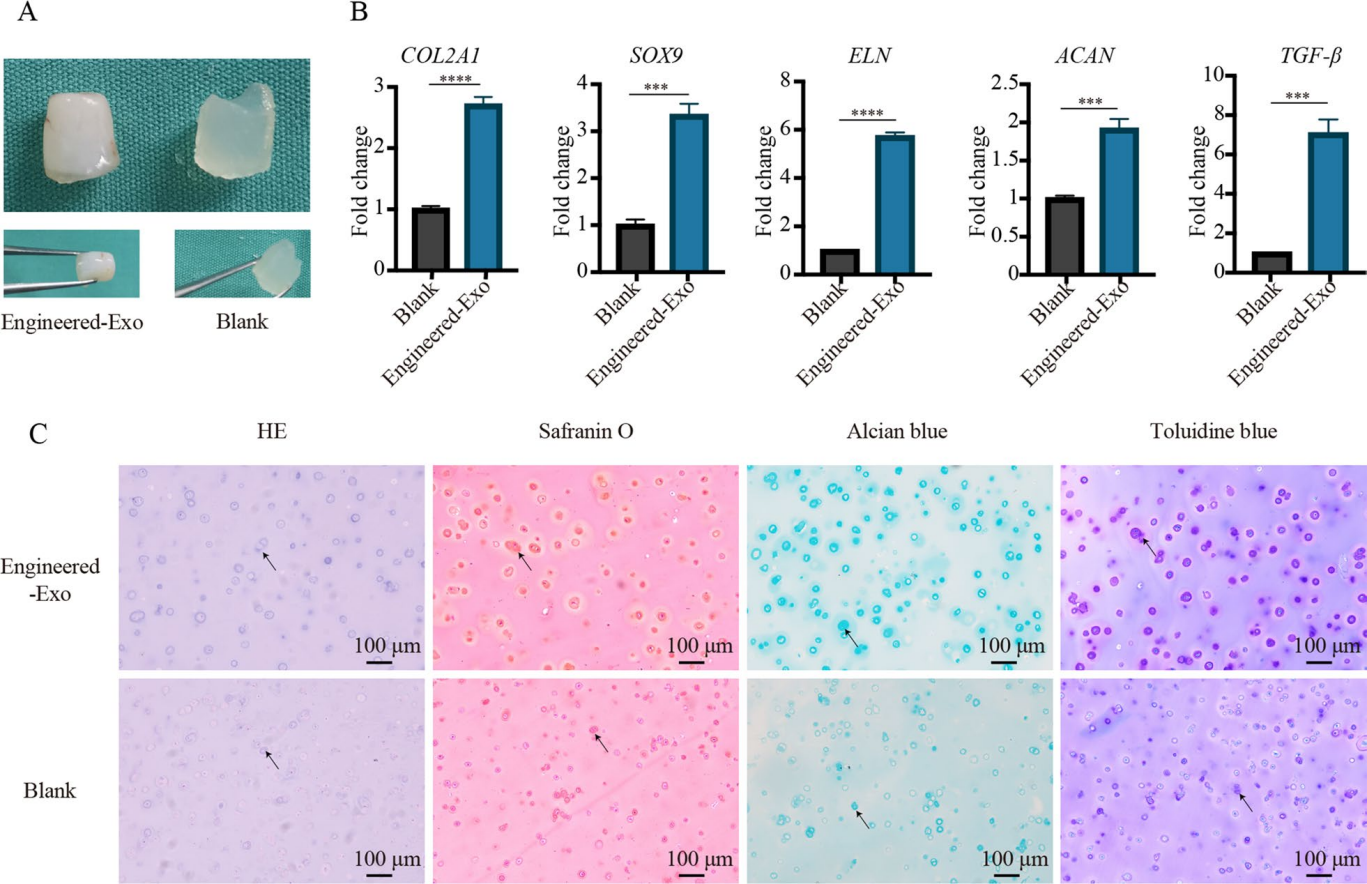
The effects of a single dose of Engineered-Exo on tissue-engineered cartilage regeneration. **A** Gross observation at 6 weeks after implantation. **B** Quantitative RT-PCR analysis showed regulation of genes at 6 weeks after implantation associated with ear cartilage matrix proteins synthesis. **C** The tissue-engineered cartilage was histologically evaluated via staining of hematoxylin and eosin (HE), Safranin O, Alcian blue and Toluidine blue. Scale bars: 100 μm. Data was presented as the mean ± SD of three number of replicates. ∗∗∗*p* < 0.001, ∗∗∗∗*p* < 0.0001

To assess the efficacy of Engineered-Exo on the survival and chondrogenesis of microtia chondrocytes/Gelma hydrogel constructs, we implanted the constructs into the subcutaneous pockets of the immunodeficient BALB/c nude mice. Gross macroscopic evaluation of the constructs was performed at 6 weeks after implantation. In Engineered-Exo group, the constructs became compact and showed ivory-white cartilaginous appearance together with a tough and elastic texture. Nevertheless, in blank control, the samples still kept weak and showed transparent hydrogel-like appearance together with a soft and fragile texture (Fig. 11A). As evaluated by qPCR, we observed that the Engineered-Exo group had a significant increase in mRNA levels of chondrogenic factor—TGF-β1 and several ear cartilage matrix proteins-COL2A1, ACAN, ELN and SOX9 in the constructs, compared to PBS control group (Fig. 11B). HE staining revealed that microtia chondrocytes were aggregated into cartilage lacuna-like structures or aggregates within Gelma hydrogel in two groups. Additionally, the secreted cartilaginous matrix (GAG) surrounding the microtia chondrocytes was significantly stained with Safranin O, Alcian blue and Toluidine blue in Engineered-Exo group (Fig. 11C). Taken together, our data demonstrate that Engineered-Exo had good potential to promote the survival and chondrogenesis of microtia chondrocytes/Gelma hydrogel constructs.

## Discussion

Tissue engineered ear is a promising alternative to external ear reconstruction, but its clinical translation has not been popularized. Our center had previously engineered patient-specific ear-shaped cartilage [5]. Although we achieved satisfactory aesthetical outcome at the first 3 years, the engineered ear gradually lost the shape and began collapsing at the long-term follow-up. One of the bottlenecks is the inadequate cell amount and poor mature cartilage formation by microtia chondrocytes. Therefore, we are making efforts to explore some trophic factors for resolving these problems.

With the potential of differentiating into chondrocytes, MSCs have proven to be efficient to repair cartilage defect [34–36]. Recently, it is increasingly thought that its cartilage repairing potential is derived from its secretion of active components, especially the extracellular vesicles/exosomes [12, 37, 38]. Exosomes are a heterogeneous group of lipid-bound nanoparticles that can deliver therapeutic molecules to specific cells, harnessing their intrinsic tissue-homing capabilities. Up to date, exosomes have been reported to act as cell-free mediators of many regenerative process in aesthetic, plastic and reconstructive surgery, such as new cartilage/bone formation [39–43], diabetic/non-diabetic wounding healing [44–47], fat grafting survival [48], angiogenesis [49, 50] and hair regeneration [51]. In the current study, we firstly isolated 3D-Exo by seeding ADSCs on porous Gelma hydrogel, and designed Engineered-Exo to increase levels of functional hsa-miR-23a-3p. The results indicated that Engineered-Exo effectively enhanced the proliferation, survival and mature cartilage formation of microtia chondrocytes via hsa-miR-23a-3p/PTEN/PI3K/AKT/mTOR axis. All in all, our study had several new findings.

Firstly, we were making technological advances towards exosomes mass production through using biomaterials-based scale-up strategy and enhancing paracrine functions of ADSCs. In our study, we found that the exosomes yield from porous Gelma hydrogel was significantly higher (3.68 to 6.64-fold) than that from 2D culture. Our result was similar to the study by Cao et al. [52] that used hollow fiber bioreactor FiberCell System and the study by Cao et al. [31] that used hydroxyapatite scaffolds for 3D culture of human umbilical cord MSCs. Actually, biomaterial scaffolds have attracted special attention in that they give the opportunity to resemble in vivo microenvironments of cells native niche, which is helpful for physiological cell-cell and cell-matrix contacts. The 3D culture conditions can optimize cell culture on a platform of limited size or scalability. It increases surface area while minimizing the volume of culture media per cell, consequently enhancing the concentration and yield of exosomes in conditioned media [15]. Gelma is one of the commonly used hydrogels simulating the physical structure and chemical composition of the natural extracellular matrix and providing a 3D template and extracellular matrix microenvironment [53]. It can be stored as lyophilized proteins and be reconstituted with culture medium prior to cell encapsulation. The porous Gelma with pores size of 100 to 200 μm was firstly used for 3D culture of ADSCs in our study. It has an appropriate swelling ratio and degradation ratio, as shown in our results, all of which can help promote cellular metabolism, cell viability and sustained exosomes release. The release kinetics curve showed that about 80% of exosomes were released in a rapid manner within 2 days. Indeed, the culture space, experimental time and expenses were greatly reduced using scale-up strategy. Recently, several studies have indicated that biomaterial scaffolds-MSCs constructs could promote the paracrine secretion of MSCs, possibly also relevant to the release of extracellular vesicles (EVs) [54–56]. We speculated that the porous characteristics of porous Gelma was beneficial to mass production of exosomes also by promoting paracrine signaling network of ADSCs. However, little is known how the biomaterials affect the paracrine effects of MSCs. To answer this question, we are investigating and comparing the effects of several kinds of hydrogels commonly used (i.e. porous Gelma, sericin methacryloyl and chondroitin sulfate methacryloyl) on exosome yield and exosomal profiles. The NTA and TEM analysis showed the successful isolation of biomaterial-based exosome (see the Additional file 15). We will analyze how the biomaterials affect the paracrine functions of ADSCs by performing proteomics and small RNA sequencing of their corresponding exosomes. We did not compare the functional effects between the 2D-Exo and 3D-Exo because the most important aim of our study was to explore the optimized exosomes for constructing tissue-engineered cartilage. Actually, the previous study by Cao et al. [31] found that 3D-Exo was superior to 2D-Exo in endothelial cell proliferation/migration/tube formation and in vivo angiogenesis. Additionally, we found that the yield of Exo 4 was significantly higher than that of Exo 2 and Exo 6, indicating that disparate passage number of ADSCs could affect the yield of their corresponding exosomes. Notably, this result was different from the study by Patel et al. [57] who found it was cell seeding density and collection frequency but not passage number that affected the yield of MSCs exosomes.

Up to date, there were no studies exploring the effects of human ADSCs passage number on the small RNA profiles and functions of their corresponding exosomes. Herein, we selected the P2, P4 and P6 ADSCs as early, middle and late cellular period for producing their corresponding exosomes. Compared with proteins and mRNA, miRNAs were more widely acknowledged as the regulatory molecules of exosomes. Small RNA sequencing results indicated that Exo 2 was mostly different from both Exo 4 and Exo 6 in terms of differential miRNAs expression. In particular, the difference was seen in several kinds of miRNAs related to new cartilage formation, such as hsa-miR-199b-5p, hsa-miR-155-5p, hsa-miR-23a-3p and hsa-miR-455-3p. KEGG enrichment pathway analysis revealed that both Exo 4 and Exo 6 consistently upregulated the PI3K-AKT and mTOR signaling pathway. Our functional assays showed that Exo 2 was more excellent than Exo 4 and Exo 6 in enhancing viability/proliferation and attenuating apoptosis of microtia chondrocytes. In contrast, both Exo 4 and Exo 6 were more efficient in promoting chondrogenesis compared to Exo 2, which was consistent with the results of small RNA sequencing. The previous study [57] found that the vascularization bioactivity of the EVs declined significantly with increasing MSCs passage number in in vitro culture, indicating that it was essential to maintain MSCs in a non-senescent state to retain the therapeutic potential of their exosomes. Notably, increased MSCs passage number attributed to alterations in genes involving cell cycle, protein ubiquitination and apoptosis, and caused the decline in proliferation ability, all of which might cause decreased cellular activity [58, 59]. In contrast, the chondrogenic differentiation capacities of P4 MSCs was significantly higher than that of earlier passage of MSCs [60, 61]. Compared to the early-passage MSCs (P3), the late-passage MSCs could stimulate chondrogenesis in co-culture with chondrocytes, indicating that MSCs had regenerative potentials through paracrine functions even after prolonging passage [62]. Taken together, though there few studies directly exploring the effects of passage number on exosomes, it could be speculated that the cellular activity of parent cells at the early, middle and late passage might affect the functions of their exosomes. On the other hand, the therapeutic potentials of MSCs might be partly reflected through their corresponding exosomes. All in all, it is quite appropriate to select P4 ADSCs for producing exosomes with the advantages of reducing parent cells senescence-related adverse events and enhancing the chondrogenesis potential of their exosomes. Notably, except for the passage number, other parameters also affected the functional contents and roles of exosomes. Li et al. [14] investigated the effects of tissue origin of MSCs on the therapeutic roles of exosomes. They found that the tissue origin contributed to the distinct protein profiles among the three types of exosomes, and ADSCs exosomes were more effective to stimulate the migration, proliferation, and chondrogenic and osteogenic differentiation of BMSCs in vitro and in vivo, compared to BMSCs exosomes and SMSCs (synovium MSCs) exosomes. Another study by Jan Van Deun et al. [7] found that disparate isolation methods for extracellular vesicles would affect the purity and downstream RNA profiling. All in all, passage number of parent ADSCs was an essential parameter for exosome-specific functional studies. That was why the minimal information for studies of extracellular vesicles 2018 (MISEV2018) guidelines [28] highlighted passage number was treated as minimal information reported in research.

Thirdly, to our knowledge, it has been reported that miR-23a-3p is related to chondrogenesis of normal or osteoarthritis (OA) chondrocytes. Park et al. [63] found that miR-23a-3p was significantly suppressed in human OA chondrocytes and overexpressing miR-23a-3p could suppressed the levels of Acyl-CoA synthetase 6 (ACSL6), caspase-1 and caspase-3, which subsequently suppressed OA pathological processes probably via upregulating type II collagen and downregulating type I collagen/metalloproteinase 13. Similarly, another study also indicated that overexpression of miR-23a-3p in normal articular chondrocytes and BMSCs could significantly promote cell proliferation/migration and enhance chondrogenesis via upregulating Aggrecan, SOX9 and type II collagen, all of which helped repair articular cartilage defect [37]. However, the inconsistent results were found that miR-23a-3p was significantly upregulated in OA chondrocytes [64, 65] and overexpression of miR-23a-3p could suppress the levels of type II collagen and aggrecan via directly targeting SMAD3 [64]. Additionally, overexpression of miR-23a-3p significantly up-regulated the expressions of IL-1β, IL-6, TNF-α, PI3K, AKT and mTOR and lowered the expressions of PTEN and IL-10, subsequently increasing the immune inflammatory response in OA patients [65]. In our study, both bioinformatic analysis and over-expression/silencing assays indicated that exosomal hsa-miR-23a-3p derived from ADSCs could suppress translation repression of PTEN and further activated the PI3K/AKT/mTOR signaling pathway, which would enhance cell viability, attenuate apoptosis and promote chondrogenesis of microtia chondrocytes. Taken together, the roles of miR-23a-3p in treating OA chondrocytes seemed to be discrepant. However, miR-23a-3p could consistently promote chondrogenesis of both normal articular chondrocytes and microtia chondrocytes under non-osteoarthritis microenvironment. Subsequently, to optimize and prolong the functional effects of ADSCs exosomes on tissue-engineered cartilage, we needed to increase level of exosomal hsa-miR-23a-3p. Actually, some of small RNA profiles from parent cells would be loaded into exosomes during the fusion of multivesicular bodies (MVBs) with the plasma membrane. Two strategies could be used to increase exosomal miRNAs either by directly transfecting functional miRNAs into exosomes or by indirectly transfecting miRNAs into parent cells to produce miRNAs-rich exosomes. Lv et al. [23] successfully applied electroporation to transfect miR-21-5p mimics into ADSCs exosomes. However, this method might damage the bi-lipid membrane structure of exosomes and lost natural availability and biocompatibility. Herein, we choose to design the genetically engineered exosomes by transfecting agomir-23a-3p to parent ADSCs and then producing natural exosomes with high-level miR-23a-3p. The Engineered-Exo showed superiority in new cartilage formation compared to the natural exosomes.

Although this study demonstrated that Engineered-Exo are efficacious in cartilage regeneration, there are still some limitations. Firstly, we need to further evaluate the effects of Engineered-Exo on the elastin composition and elasticity characteristics of the tissue-engineered cartilage. Microtia chondrocytes, which is isolated from the patient’s microtia cartilage without injuring healthy ear or cartilage of other sites, are a promising cell source due to their abilities to form elastic cartilage. In our study, we firstly found that Engineered-Exo enhanced the mRNA and protein levels of elastin, a key intercellular composition for increasing ear cartilage elasticity. Now, we are using 3D bio-printing to directly fabricate ear-shaped microtia chondrocytes/Gelma hydrogel/Engineered-Exo constructs, which will be implanted into the subcutaneous pockets of nude mice for up to 4 months to investigate the long-term effects of Engineered-Exo on engineered elastic cartilage. The mechanical measurement is essential for the assessment of the long-term stability of the engineered elastic cartilage. Therefore, the Young’s modulus, final relaxation slope, and relaxation amout of these constructs will be analyzed through tensile and compressive tests using a material testing machine. A modified Verhoeff van Gieson (EvG) elastic staining will be performed to detect the levels of intercellular elastin. The above characterization of the constructs will be compared versus native ear cartilage to seek common ground while shelving differences. Secondly, we only confirm the functional roles of exosomal hsa-miR-23a-3p for microtia chondrocytes. Nevertheless, there are multiple exosomal miRNAs related to chondrogenesis detected in the results of small RNA sequencing, such as hsa-mir-199b-5p, hsa-mir-155-5p and hsa-mir-455-3p (Fig. 5B). That is, ADSCs exosomes affect microtia chondrocytes probably through multiple underlying mechanisms. Therefore, in further studies, it would be necessary to explore the integrated regulatory mechanisms. Thirdly, the minimal effective dose of Engineered-Exo needs to be optimized for tissue-engineered cartilage, probably by reducing the dose and increasing times. Furthermore, the release ratio of exosomes should be further enhanced and the degradation of porous Gelma ratio should be further postponed for the continuous and steady collection of exosomes. Lastly, to generate ear-shaped cartilage with pre-designed 3D structure, the unfavorable host response to the engineered graft after its transplantation needed to resolved. Whether the use of Engineered-exosomes could decrease host response deserves further research.

## Conclusions

We successfully performed mass production of 3D-Exo using porous Gelma hydrogel. The 3D-Exo derived from disparate passage number of ADSCs showed distinction in small RNA profiles and functions. We optimized exosomes by selecting the P4 ADSCs as parent cells and designing Engineered-Exo with high content of hsa-mir-23a-3p. A single dose of Engineered-Exo was efficacious for tissue-engineered cartilage regeneration.

## Supplementary Information

**Additional file 1.** Supplementary materials and methods.

**Additional file 2.** The essential parameters for 2D/3D exosomes production and the comparison of yield.

**Additional file 3.** The details of differential ultracentrifuge for exosomes isolation.

**Additional file 4.** Primer sequences used for quantitative RT-PCR.

**Additional file 5.** Antibodies used for western blotting.

**Additional file 6.** The original western blotting images for Fig. 2C.

**Additional file 7.** Experiment design for in vitro studies.

**Additional file 8.** Bioinformatics analysis. (**A**) The potential 156 target genes of hsa-miR-23a-3p. (**B**) The potential target sequences of hsa-miR-23a-3p.

**Additional file 9.** The original western blotting images for Fig. 7C.

**Additional file 10.** The original western blotting images for Fig. 8E.

**Additional file 11.** The original western blotting images for Fig. 8F.

**Additional file 12.** The original western blooting images for Fig. 9C.

**Additional file 13.** The original western blooting images for Fig. 9E.

**Additional file 14.** The original western blooting images for Fig. 10F.

**Additional file 15.** Identification of ADSCs-Exo isolated from conditioned medium of biomaterials-based 3D culture (PG-Exo, PG + ChsMa-Exo, and PG + SerMa-Exo) and 2D culture (2D-Exo). (**A**) Morphological characterization via transmission electron microscopy. (**B**) Particle size distribution measured using nanoparticle tracking analysis. Scale bar:100 nm. PG: porous GelMa, PG + ChsMa: porous GelMa + chondroitin sulfate methacryloyl, PG + SerMa: porous GelMa + sericin methacryloyl.

## Data Availability

All data generated or analyzed during this study are included in this published article.
