# Supplementary material for: Congenital microtia patients: the genetically engineered exosomes released from porous gelatin methacryloyl hydrogel for downstream small RNA profiling, functional modulation of microtia chondrocytes and tissue-engineered ear cartilage regeneration
Source: J Nanobiotechnology. 2022 Mar 28;20:164. doi: 10.1186/s12951-022-01352-6 (PMC8962601; doi:10.1186/s12951-022-01352-6)
Supplement: Supplementary file 1 — Additional file 1. Supplementary materials and methods. [file 12951_2022_1352_MOESM1_ESM.docx]

**1. Supplementary materials and methods**

***1.1. Cell isolation and culture***

The microtia cartilage was cut into pieces (1 to 2 mm^3^) and digested with 0.2% type IV collagenase (Sigma‐Aldrich, C5138-1G) overnight at 37°C with continuous shaking. The microtia chondrocytes were separated and cultured in a humidified 37°C and 5% CO_2_ incubator. DMEM medium (Gibco, 10566016) was supplemented with 10% FBS (Gibco, 10099141), 100 U/ml penicillin and 100 μg/ml streptomycin (Gibco, 15140122). Microtia chondrocytes cultured to the second passage were used in our study.

Briefly, the inguinal adipose tissues from the right chest wall were finely minced and digested in phosphate-buffered saline (PBS) (Gibco, 20012050) containing 0.1% type I collagenase (Sigma‐Aldrich, C0130-1G) for 45 minutes at 37 °C with continuous shaking. After centrifugation, the top lipid layers were removed, and the primary cells were re-suspended and cultured in mesenchymal stem cell medium (ScienCell, 7501) with 500 ml of basal medium, 25 ml of fetal bovine serum (ScienCell, 0025), 5 ml of mesenchymal stem cell growth supplement (ScienCell, 7552) and 5 ml of penicillin/streptomycin solution (ScienCell, 0503).

***1.2. The synthesis of porous Gelma hydrogel composites***

With pores size of 100 to 200 nm, porous Gelma hydrogels (Engineering for Life of Yongqinquan, Suzhou, China) were synthesized as follows: porous Gelma was firstly dissolved in PBS with a concentration of 6% w/v, and then fully mixed by magnetic stirring. The composited hydrogel was obtained after photo-crosslinking using a blue laser with a short-wave length 405nm for 18 s.

***1.3 Cellular uptake of exosomes***

Briefly, exosomes were incubated with 500 ul dilution C solution and 4ul PKH-26 dye solution for 5 min at room temperature in dark environment. Then, 500 ul 1% bovine serum albumin were added to stop the process of staining. The labelled exosomes were obtained after be centrifuged twice at 100,000 g for 70 min and resuspended with 100 ul cold PBS.

Briefly, microtia chondrocytes were incubated with 5μM working solution of CFDA SE for 10 min at room temperature in dark environment. Then, same volume of FBS were added to stop the process of staining. The labelled chondrocytes were washed and centrifuged twice, and were resuspended at culture dish for 2D culture or mixed with Gelma hydrogel for 3D culture.

***1.4. Small RNA sequencing and analysis***

According to manufacturer’s recommendations, sequencing libraries were generated using NEBNext®Multiplex Small RNA Library Prep Set for Illumina® (NEB, USA.) and index codes were added to attribute sequences to each sample. The library preparations were sequenced on an Illumina Hiseq 2500/2000 platform and 50bp single-end reads were generated. The differential expression analysis was performed using the DESeq R package (3.0.3). The P-values was adjusted using the Benjamini& Hochberg method and corrected P value of 0.05 was set as the threshold for significantly differential expression by default.
