# Supplementary material for: Congenital microtia patients: the genetically engineered exosomes released from porous gelatin methacryloyl hydrogel for downstream small RNA profiling, functional modulation of microtia chondrocytes and tissue-engineered ear cartilage regeneration
Source: J Nanobiotechnology. 2022 Mar 28;20:164. doi: 10.1186/s12951-022-01352-6 (PMC8962601; doi:10.1186/s12951-022-01352-6)
Supplement: Supplementary file 2 — Additional file 2. The essential parameters for 2D/3D exosomes production and the comparison of yield. [file 12951_2022_1352_MOESM2_ESM.docx]

| Exo | Culture technique | Cell passage (P) | Cells seeding | Medium, serum | Collection time | Isolation method | Volume processes | Protein yield (μg/10 ml) |
| --- | --- | --- | --- | --- | --- | --- | --- | --- |
| 2D-Exo 2 | 2 D culture dish | P2 | 1E6 in 10 ml medium | MSCM, exo-free serum | 2 days | UC | 10 | 18.66 to 23.90 |
| 2D-Exo 4 | 2 D culture dish | P4 | 1E6 in 10 ml medium | MSCM, exo-free serum | 2 days | UC | 10 | 21.27 to 26.74 |
| 2D-Exo 6 | 2 D culture dish | P6 | 1E6 in 10 ml medium | MSCM, exo-free serum | 2 days | UC | 10 | 17.53 to 24.43 |
| 3D-Exo 2 | Porous GelMA | P2 | 1E6 in 100 ul porous GelMA in 5 ml medium | MSCM, exo-free serum | 2 days | UC | 10 | 79.39 to 82.56 |
| 3D-Exo 4 | Porous GelMA | P4 | 1E6 in 100 ul porous GelMA in 5 ml medium | MSCM, exo-free serum | 2 days | UC | 10 | 160.19 to 167.71 |
| 3D-Exo 6 | Porous GelMA | P6 | 1E6 in 100 ul porous GelMA in 5 ml medium | MSCM, exo-free serum | 2 days | UC | 10 | 109.77 to 110.06 |

Additional file 2: The essential parameters for 2D/3D exosomes production and the comparison of yield

Exo: exosomes; 2D: two dimensional; 3D: three dimensional; MSCM: mesenchymal stem cell medium; UC: ultracentrifugation
