# Supplementary figures and images for: Congenital microtia patients: the genetically engineered exosomes released from porous gelatin methacryloyl hydrogel for downstream small RNA profiling, functional modulation of microtia chondrocytes and tissue-engineered ear cartilage regeneration
Source: J Nanobiotechnology. 2022 Mar 28;20:164. doi: 10.1186/s12951-022-01352-6 (PMC8962601; doi:10.1186/s12951-022-01352-6)

Figure caption: The details of differential ultracentrifuge for exosomes isolation.

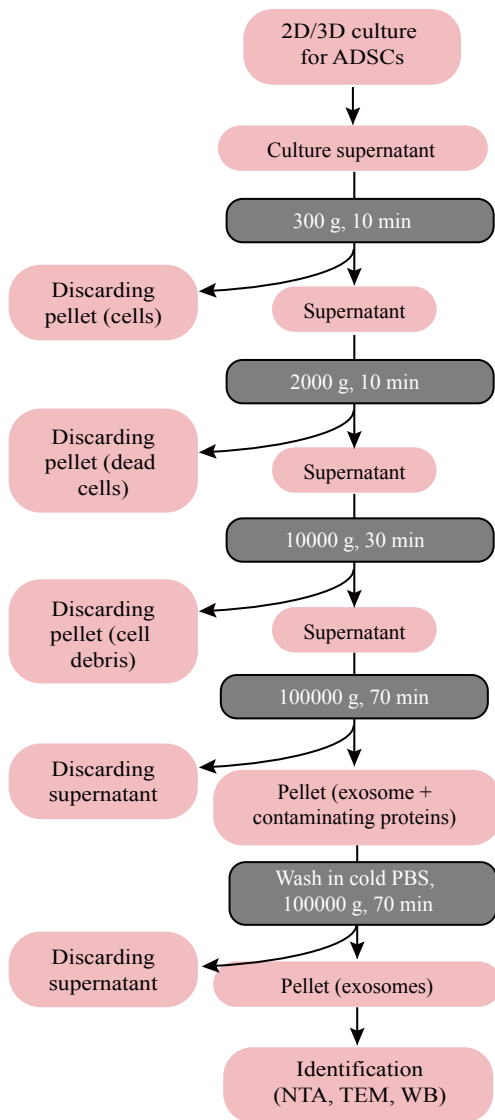

Supplement: Supplementary file 3 — Additional file 3. The details of differential ultracentrifuge for exosomes isolation. [file 12951_2022_1352_MOESM3_ESM.pdf]

Figure caption: The original western blotting images

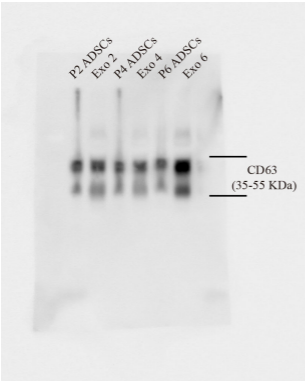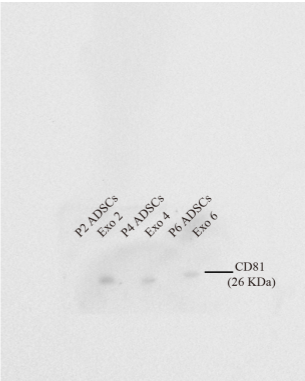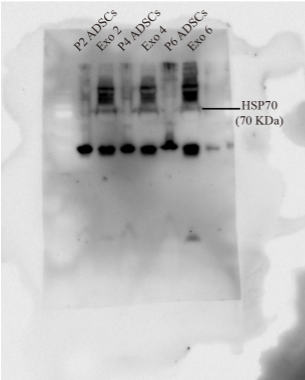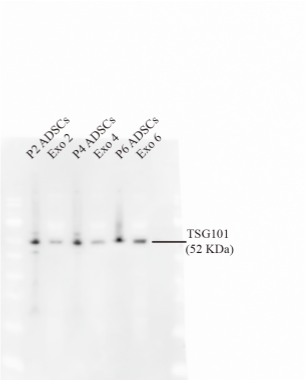

Supplement: Supplementary file 6 — Additional file 6. The original western blotting images for Fig. 2C. [file 12951_2022_1352_MOESM6_ESM.pdf]

Figure caption: The original western blotting images

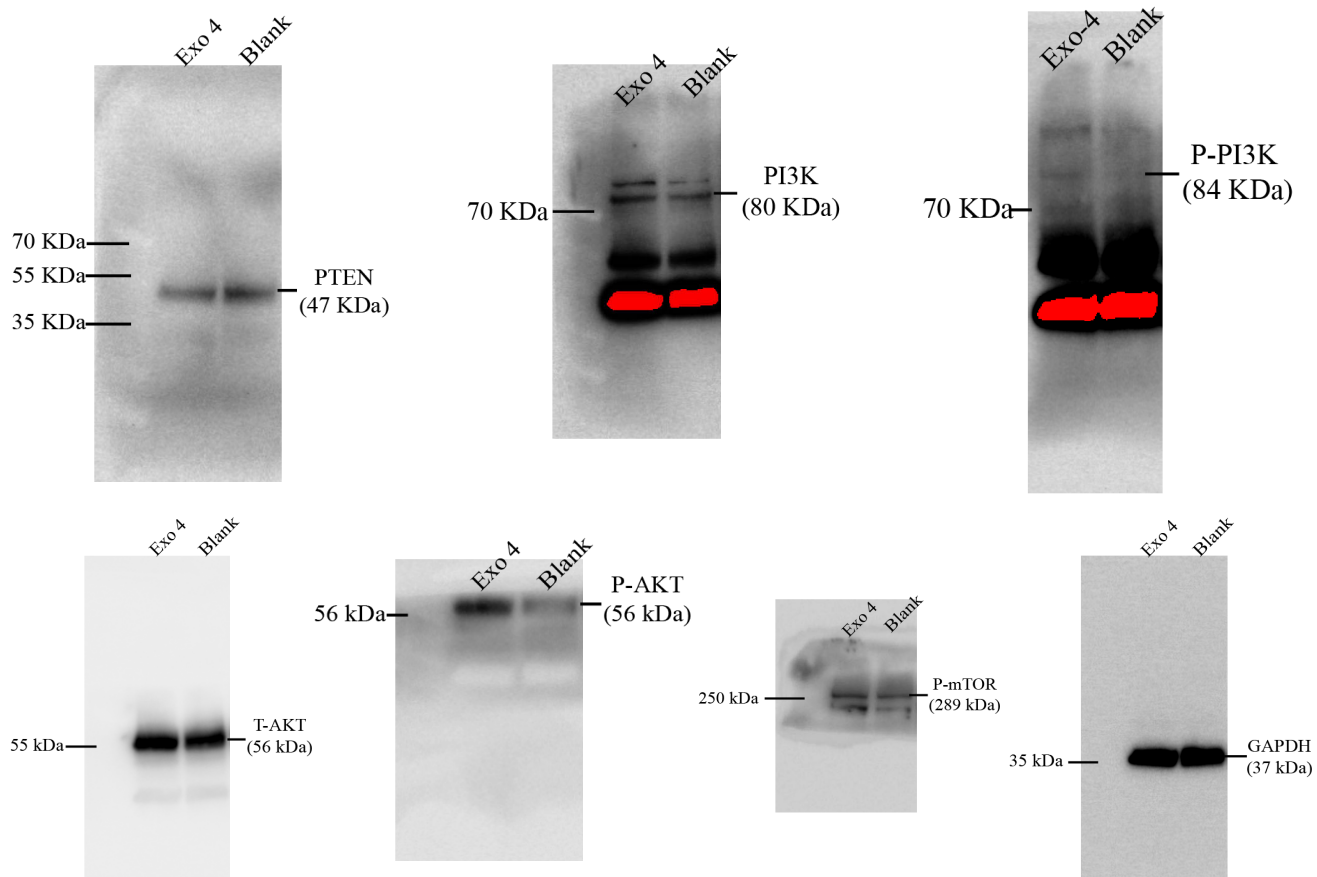

Supplement: Supplementary file 9 — Additional file 9. The original western blotting images for Fig. 7C. [file 12951_2022_1352_MOESM9_ESM.pdf]

Figure caption: The original western blotting images

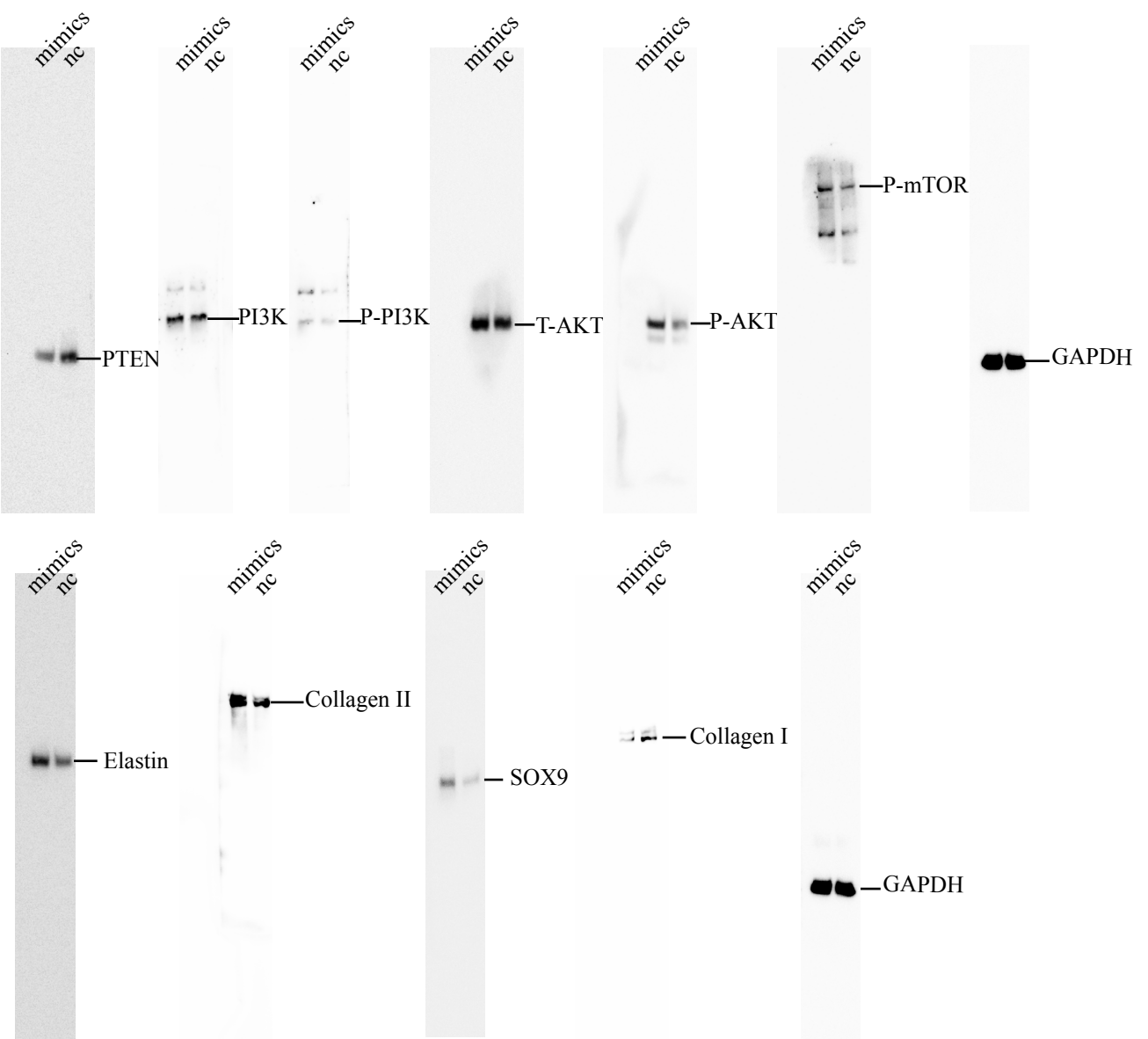

Supplement: Supplementary file 10 — Additional file 10. The original western blotting images for Fig. 8E. [file 12951_2022_1352_MOESM10_ESM.pdf]

Figure caption: The original western blotting images

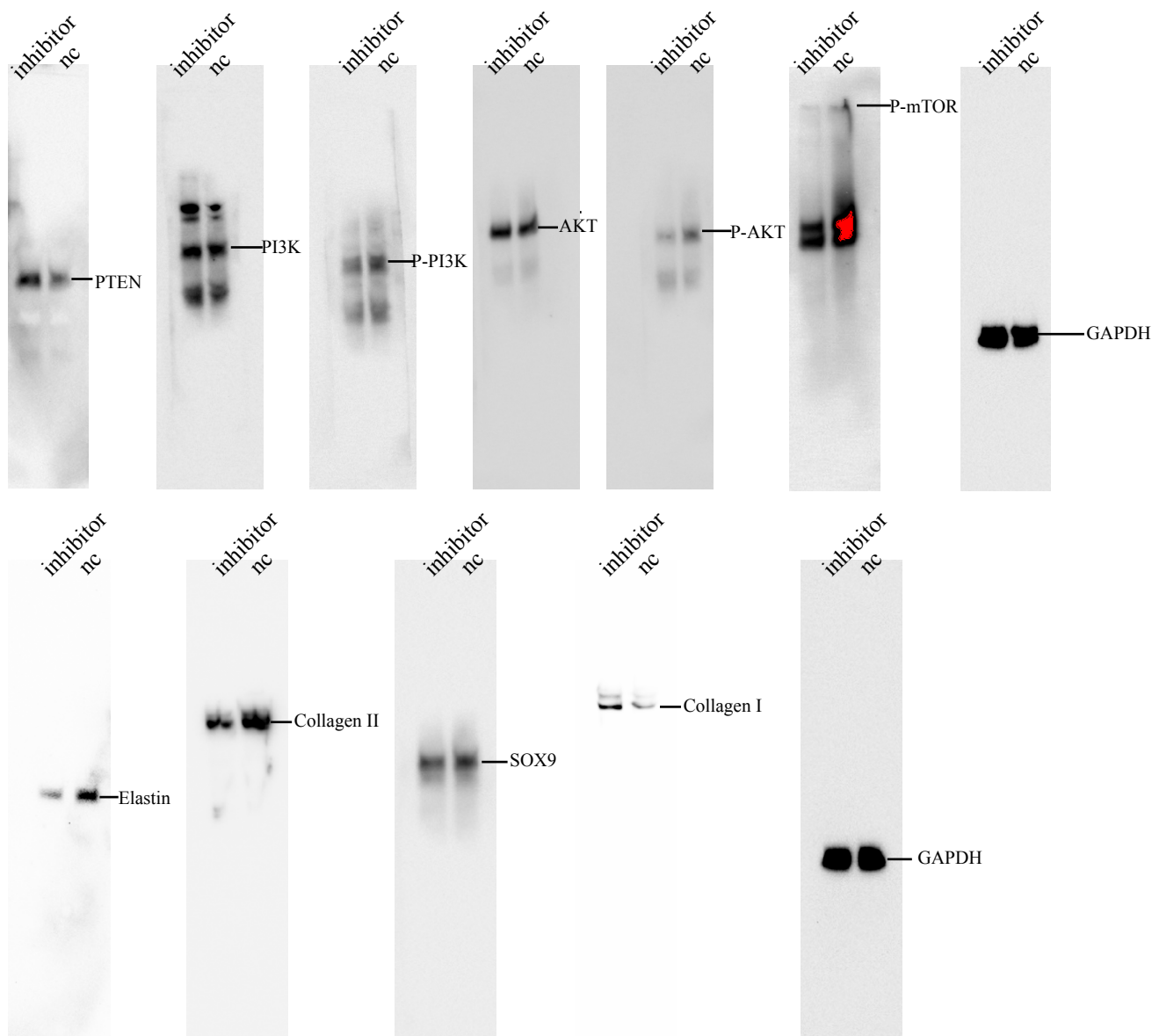

Supplement: Supplementary file 11 — Additional file 11. The original western blotting images for Fig. 8F. [file 12951_2022_1352_MOESM11_ESM.pdf]

Figure caption: The original western blotting images

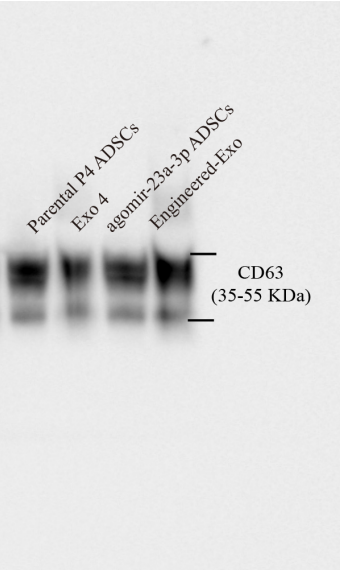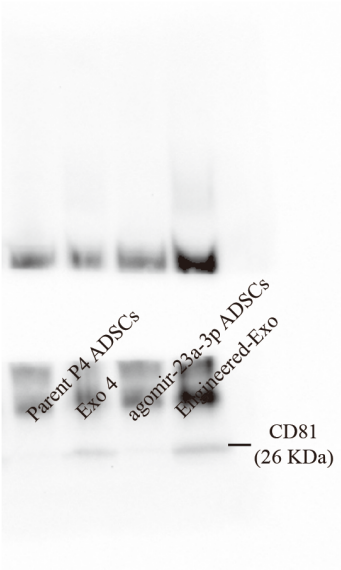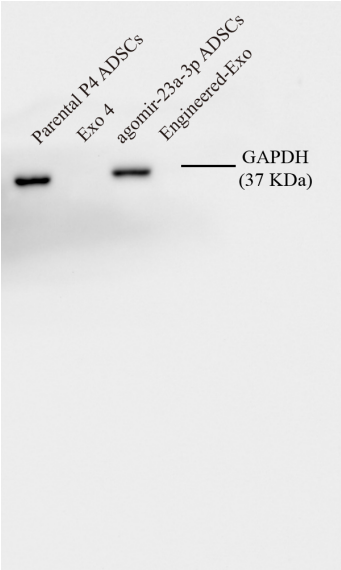

Supplement: Supplementary file 12 — Additional file 12. The original western blooting images for Fig. 9C. [file 12951_2022_1352_MOESM12_ESM.pdf]

Figure caption: The original western blotting images

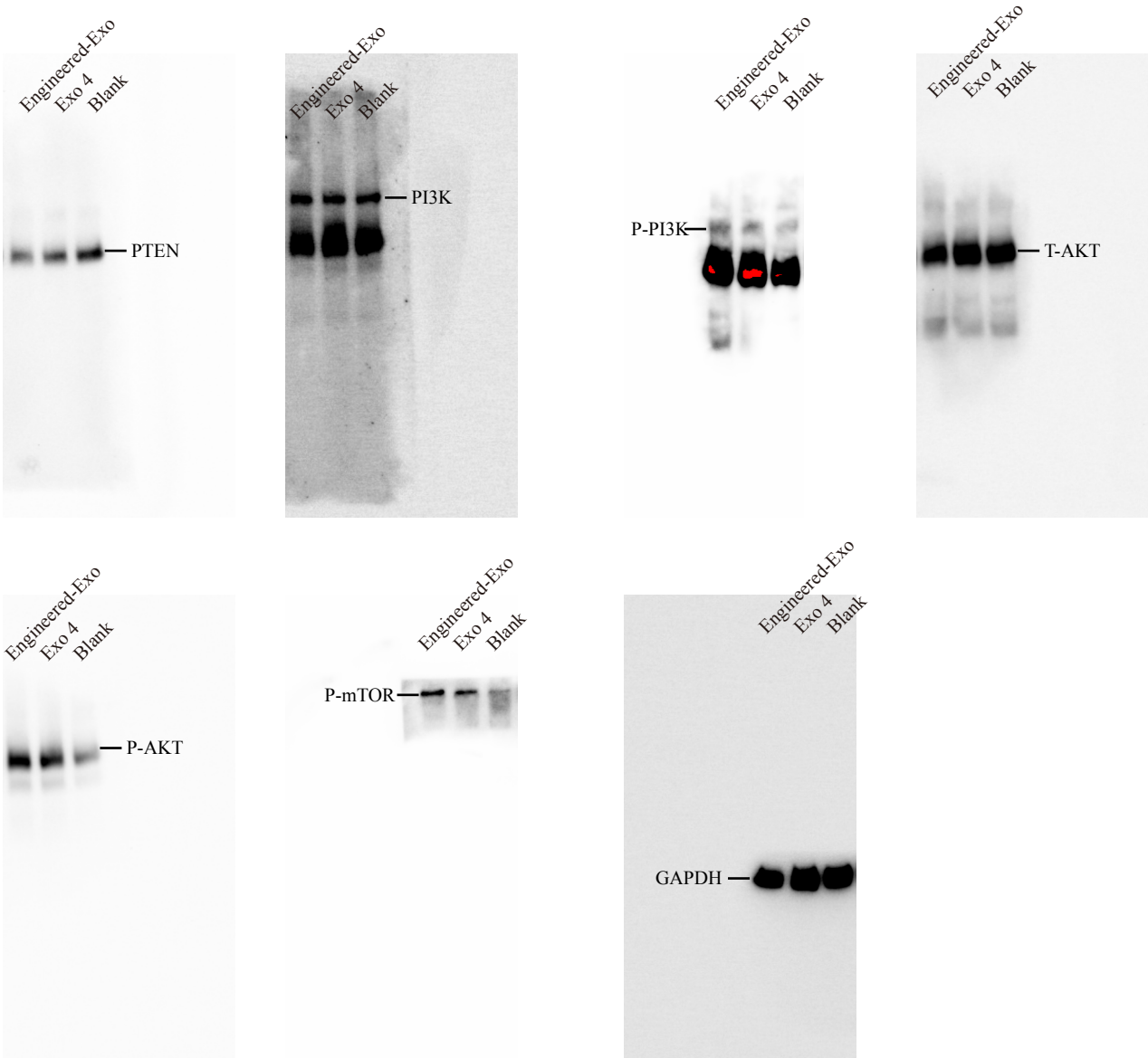

Supplement: Supplementary file 13 — Additional file 13. The original western blooting images for Fig. 9E. [file 12951_2022_1352_MOESM13_ESM.pdf]

Additional file 14: The original western blotting

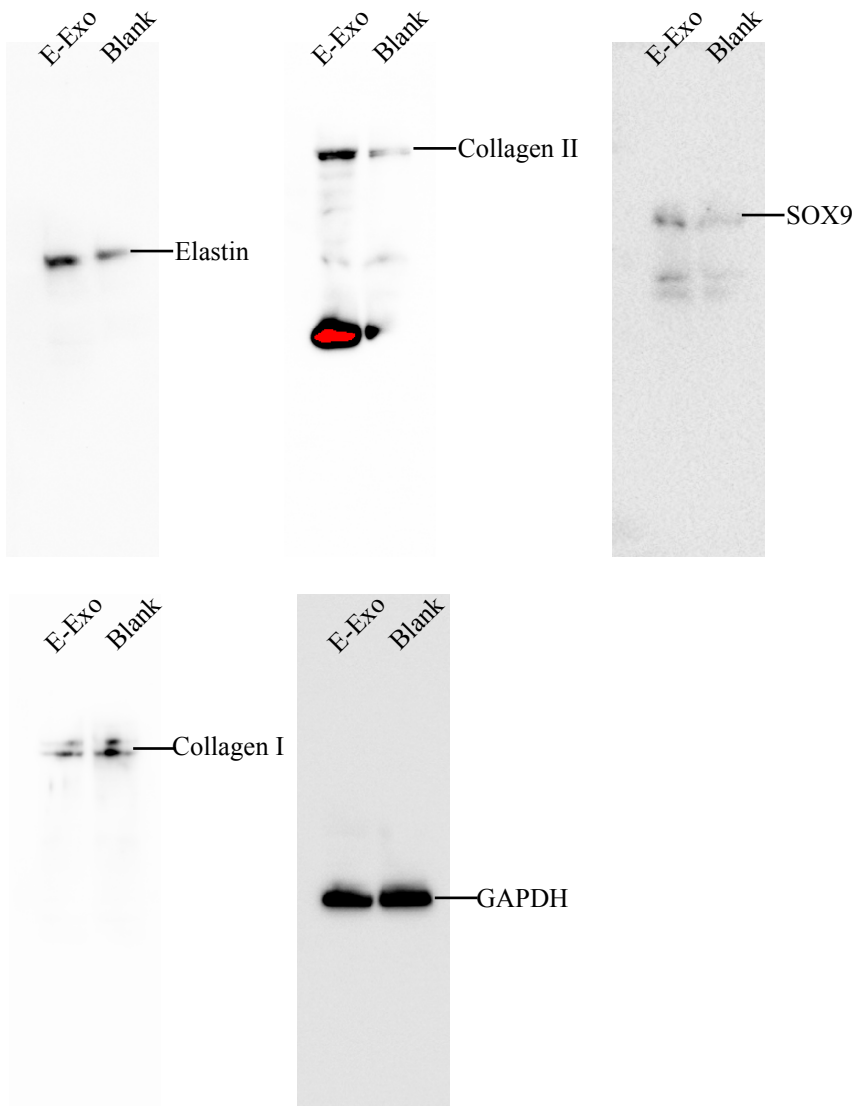

Supplement: Supplementary file 14 — Additional file 14. The original western blooting images for Fig. 10F. [file 12951_2022_1352_MOESM14_ESM.pdf]
