# Supplementary material for: Congenital microtia patients: the genetically engineered exosomes released from porous gelatin methacryloyl hydrogel for downstream small RNA profiling, functional modulation of microtia chondrocytes and tissue-engineered ear cartilage regeneration
Source: J Nanobiotechnology. 2022 Mar 28;20:164. doi: 10.1186/s12951-022-01352-6 (PMC8962601; doi:10.1186/s12951-022-01352-6)
Supplement: Supplementary file 4 — Additional file 4. Primer sequences used for quantitative RT-PCR. [file 12951_2022_1352_MOESM4_ESM.doc]

| Gene | Primer sequence (5' to 3') |
| --- | --- |
| *SOX9* | **F:** AGCGAACGCACATCAAGAC, **R:** CTGTAGGCGATCTGTTGGGG |
| *ACAN* | **F:** ACTCTGGGTTTTCGTGACTCT, **R:** ACACTCAGCGAGTTGTCATGG |
| *COL2A1* | **F:** TGGACGCCATGAAGGTTTTCT, **R:** TGGGAGCCAGATTGTCATCTC |
| *COMP* | **F:** GATCACGTTCCTGAAAAACACG, **R:** GCTCTCCGTCTGGATGCAG |
| *ELN* | **F:** GCAGGAGTTAAGCCCAAGG, **R:** TGTAGGGCAGTCCATAGCCA |
| *COL1A1* | **F:** GAGGGCCAAGACGAAGACATC, **R:** CAGATCACGTCATCGCACAAC |
| *PTEN* | **F:** TTTGAAGACCATAACCCACCAC, **R:** ATTACACCAGTTCGTCCCTTTC |
| *TGF-beta 1* | **F:** CAATTCCTGGCGATACCTCAG, **R:** GCACAACTCCGGTGACATCAA |
| *Bcl-2* | **F:** GGTGGGGTCATGTGTGTGG, **R:** CGGTTCAGGTACTCAGTCATCC |
| *PCNA* | **F:** CCTGCTGGGATATTAGCTCCA, **R:** CAGCGGTAGGTGTCGAAGC |
| *FGF-2* | **F:** AGAAGAGCGACCCTCACATCA, **R:** CGGTTAGCACACACTCCTTTG |
| *Survivin* | **F:** AGGACCACCGCATCTCTACAT, **R:** AAGTCTGGCTCGTTCTCAGTG |
| *MMP9* | **F:** AGACCTGGGCAGATTCCAAAC, **R:** CGGCAAGTCTTCCGAGTAGT |
| *hsa−miR−23a−3p* | **F:** GCGATCACATTGCCAGGG, **R:** AGTGCAGGGTCCGAGGTATT  **RT:** GTCGTATCCAGTGCAGGGTCCGAGGTATTCGCACTGGATACGACGGAAAT |
| *5S* | **F:** GCCATACCACCCTGAACGC, **R:** CGGTATTCCCAGGCGGTCT |
| *GAPDH* | **F:** GGAGCGAGATCCCTCCAAAAT, **R:** GGCTGTTGTCATACTTCTCATGG |

Additional files 4: Primer sequences used for quantitative RT-PCR.
