# Supplementary material for: Congenital microtia patients: the genetically engineered exosomes released from porous gelatin methacryloyl hydrogel for downstream small RNA profiling, functional modulation of microtia chondrocytes and tissue-engineered ear cartilage regeneration
Source: J Nanobiotechnology. 2022 Mar 28;20:164. doi: 10.1186/s12951-022-01352-6 (PMC8962601; doi:10.1186/s12951-022-01352-6)
Supplement: Supplementary file 5 — Additional file 5. Antibodies used for western blotting. [file 12951_2022_1352_MOESM5_ESM.doc]

Additional file 5: Antibodies used for western blotting

| Antibody | Clone | Dilution | Company |
| --- | --- | --- | --- |
| CD63 | Rabbit monoclonal | 1:1000 | abcam |
| CD81 | Rabbit monoclonal | 1:1000 | abcam |
| TSG101 | Rabbit monoclonal | 1:1000 | abcam |
| HSP70 | Rabbit monoclonal | 1:1000 | abcam |
| PTEN | Rabbit monoclonal | 1:1000 | abcam |
| PI3K | Rabbit monoclonal | 1:1000 | abcam |
| P-PI3K | Rabbit polyclonal | 1:500 | abcam |
| AKT | Rabbit monoclonal | 1:10000 | abcam |
| P-AKT | Rabbit monoclonal | 1:1000 | abcam |
| P-mTOR | Rabbit monoclonal | 1:1000 | abcam |
| Type I collagen | Rabbit monoclonal | 1:1000 | abcam |
| SOX9 | Rabbit monoclonal | 1:1000 | abcam |
| Elastin | Rabbit monoclonal | 1:1000 | abcam |
| Type II collagen | Rabbit polyclonal | 1:1000 | Invitrogen |
| GAPDH | Mouse polyclonal | 1:3000 | Huaxingbio |
