# Supplementary material for: Congenital microtia patients: the genetically engineered exosomes released from porous gelatin methacryloyl hydrogel for downstream small RNA profiling, functional modulation of microtia chondrocytes and tissue-engineered ear cartilage regeneration
Source: J Nanobiotechnology. 2022 Mar 28;20:164. doi: 10.1186/s12951-022-01352-6 (PMC8962601; doi:10.1186/s12951-022-01352-6)

Figure caption: Experiment design for in vitro studies

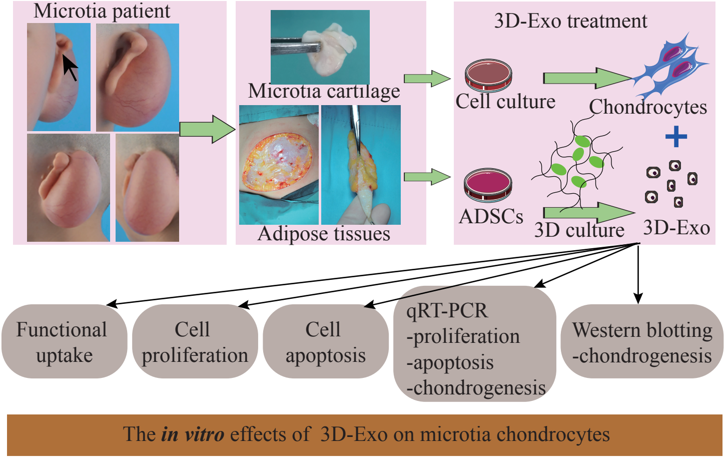

Supplement: Supplementary file 7 — Additional file 7. Experiment design for in vitro studies. [file 12951_2022_1352_MOESM7_ESM.pdf]
