# Supplementary material for: Congenital microtia patients: the genetically engineered exosomes released from porous gelatin methacryloyl hydrogel for downstream small RNA profiling, functional modulation of microtia chondrocytes and tissue-engineered ear cartilage regeneration
Source: J Nanobiotechnology. 2022 Mar 28;20:164. doi: 10.1186/s12951-022-01352-6 (PMC8962601; doi:10.1186/s12951-022-01352-6)

Figure caption: Bioinformatics analysis. (A) The potential 156 target genes of hsa-miR-23a-3p. (B) The potential target sequences of hsa-miR-23a-3p.

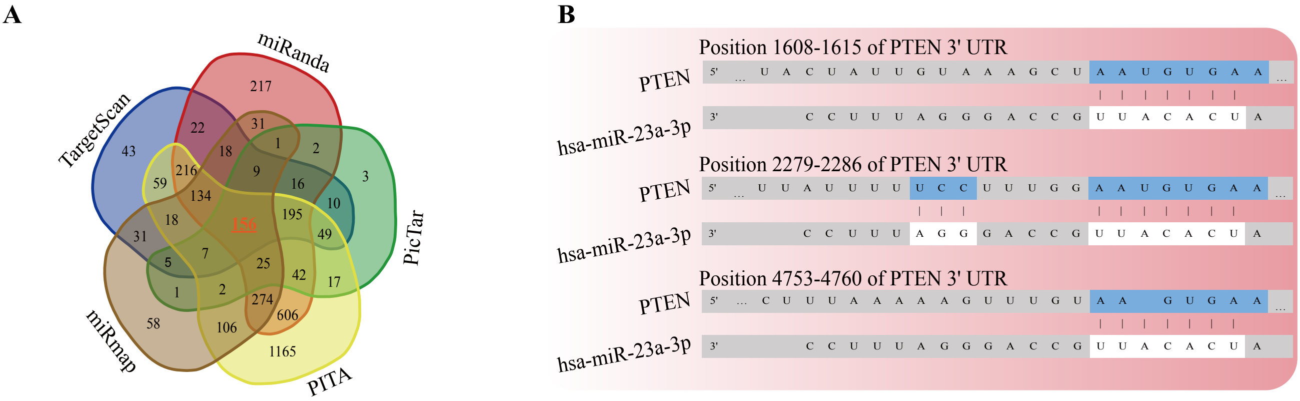

Supplement: Supplementary file 8 — Additional file 8. Bioinformatics analysis. (A) The potential 156 target genes of hsa-miR-23a-3p. (B) The potential target sequences of hsa-miR-23a-3p. [file 12951_2022_1352_MOESM8_ESM.pdf]
