# Supplementary material for: Congenital microtia patients: the genetically engineered exosomes released from porous gelatin methacryloyl hydrogel for downstream small RNA profiling, functional modulation of microtia chondrocytes and tissue-engineered ear cartilage regeneration
Source: J Nanobiotechnology. 2022 Mar 28;20:164. doi: 10.1186/s12951-022-01352-6 (PMC8962601; doi:10.1186/s12951-022-01352-6)

Figure caption: Identification of ADSCs-Exo isolated from conditioned medium of biomaterials-based 3D culture (PG-Exo, PG + ChsMa-Exo, and PG + SerMa-Exo) and 2D culture (2D-Exo). (A) Morphological characterization via transmission electron microscopy. (B) Particle size distribution measured using nanoparticle tracking analysis. Scale bar:100 nm. PG: porous GelMa, PG + ChsMa: porous GelMa + chondroitin sulfate methacryloyl, PG + SerMa: porous GelMa + sericin methacryloyl.

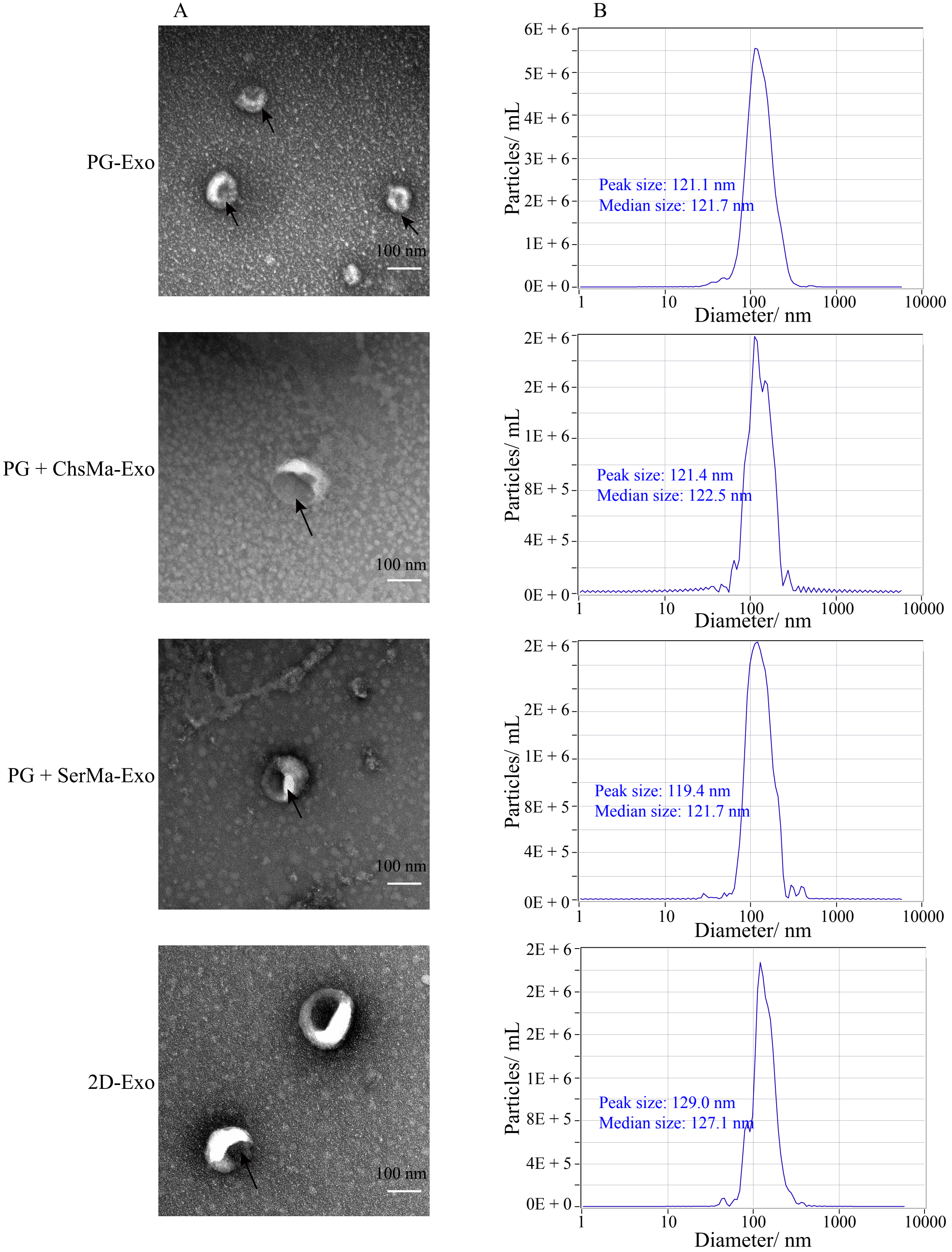

Supplement: Supplementary file 15 — Additional file 15. Identification of ADSCs-Exo isolated from conditioned medium of biomaterials-based 3D culture (PG-Exo, PG + ChsMa-Exo, and PG + SerMa-Exo) and 2D culture (2D-Exo). (A) Morphological characterization via transmission electron microscopy. (B) Particle size distribution measured using nanoparticle tracking analysis. Scale bar:100 nm. PG: porous GelMa, PG + ChsMa: porous GelMa + chondroitin sulfate methacryloyl, PG + SerMa: porous GelMa + sericin methacryloyl. [file 12951_2022_1352_MOESM15_ESM.pdf]
